# Supplementary material for: Chalcogen-bridged coordination polymer for the photocatalytic activation of aryl halides
Source: Nat Commun. 2023 Jul 6;14:4002. doi: 10.1038/s41467-023-39540-z (PMC10326065; doi:10.1038/s41467-023-39540-z)
Supplement: Supplementary file 1 — Supplementary Information [file 41467_2023_39540_MOESM1_ESM.pdf]

Supplementary Information

Chalcogen-bridged coordination polymer for the photocatalytic activation of aryl halides

Le Zeng[1‡], Tiexin Zhang[1‡*], Renhai Liu[1‡], Wenming Tian[2], Kaifeng Wu[2], Jingyi Zhu[2], Zhonghe Wang[1], Cheng He[1], Jing Feng[1], Xiangyang Guo[2], Abdoulkader Ibro Douka[1], & Chunying Duan[1*]

[1] State Key Laboratory of Fine Chemicals, School of Chemical Engineering, School of Chemistry, Dalian University of Technology, Dalian, 116024.

[2] Dalian Institute of Chemical Physics, Chinese Academy of Sciences, Dalian, 116023.

Email: zhangtiexin@dlut.edu.cn; cyduan@dlut.edu.cn

Contents

Experimental Details for Figures in Manuscript 2
Supplementary Demonstration of Dye-Aggregation Improved PET for Inert Bond Cleavage 4
Syntheses of NDI-based Coordination Polymers 5
Supplementary Structural and Photoelectronic Tables and Figures of Coordination Polymers 7
Supplementary Experimental Methods and Analyses of Transient Absorption Spectra 15
Supplementary Experimental Methods of Transient Photocurrent Responses 17
Typical Procedure for the Photocleavage of Aryl Halides 17
Characterisation of Tandem Photocleavage and C–C and C–X Bond Formation Reactions 23
^{1}H and ^{13}C NMR spectra of the isolated compounds 26
References 37

Experimental Details for Figures in Manuscript

For Fig. 2a, the original curves of UV-vis and photoluminescence were from Figs. 3a, 3b, and Fig. 2c, respectively, which were subjected to normalization.

For Figs. 2b and 8a, please refer to Materials and measurements, Manuscript for the experimental procedures of differential pulse voltammetry (DPV) spectra.

For Fig. 2c, the DMF stock solution of H_4SNDI was prepared by adding 3.6 mg H_4SNDI (*ca*. 0.005 mmol) to 2 mL DMF, and the DMF suspension of Cd–SNDI was prepared by finely grinding and sonicating the mixture of *ca*. 5.3 mg Cd–SNDI (containing *ca*. 0.005 mmol NDI moieties) and 2 mL DMF. After several iterative rounds of tentatively scanning the emission and excitation spectra, 405 nm was chosen as the common excitation wavelength for both the H_4SNDI solution and the Cd–SNDI suspension. By diluting the stock solution of H_4SNDI and the suspension of Cd–SNDI, their absorbances at *ca*. 405 nm were adjusted to the same level before further examination on photoluminescence, as shown in Fig. 2c. Then, the samples of H_4SNDI and Cd–SNDI were subjected to the examinations of photoluminescence lifetime, as shown in Fig. 2d.

For Figs. 2e and 2f, please refer to Supplementary Figs. 8a and 8b and related figure captions for details of comparative electron paramagnetic resonance (EPR) spectra.

For Fig. 3a, the DMF stock solution of H_4SNDI was prepared by adding 3.6 mg H_4SNDI to 2 mL DMF. The concentration for the tested solution was diluted to make the absorption at 360 nm around 1.0, and the solution was devoted to the first UV-vis analysis. Then, the excess amount of 56 μL Et_3N was added to the solution of H_4SNDI, which turned the orange solution to red colour, and the solution was devoted to the second UV-vis analysis. The mixed solution was then purged with N_2 for 5 min and followed by 10 min of irradiation by a 455 nm LED. The resulting solution exhibited a dark-purple color, and was devoted to the third UV-vis analysis.

For Fig. 3b, the DMF suspension of Cd–SNDI was prepared by finely grinding and sonicating the mixture of *ca*. 5.3 mg Cd–SNDI and 2 mL DMF. The concentration for the tested suspension was diluted to make the absorption at the range of 360~370 nm around 1.0, and the suspension was devoted to the first UV-vis analysis. Then, the excess amount of 56 μL Et_3N was added to the suspension of Cd–SNDI for the second UV-vis analysis. The suspension was purged with N_2 for 5 min, followed by 10 min of irradiation from a 455 nm LED, and was devoted to the third UV-vis analysis.

For Fig. 3c, the DMF suspension of Cd–SNDI was prepared by finely grinding and sonicating the mixture of *ca*. 5.3 mg Cd–SNDI and 2 mL DMF. The concentration for the suspension was diluted to make the absorption at the range of 360~370 nm around 1.0. Then, the excess amount of 56 μL Et_3N was added, and the suspension was purged with N_2 for 5 min, followed by 10 min of irradiation from a 455 nm LED. 25 μL stock solution (0.1 M in DMF) of 4'-chloroacetophenone **1a** (*ca*. 0.0025 mmol) was added to the above suspension for UV-vis analysis. And another 25 μL stock solution of **1a** was added for the successive UV-vis analysis. Then, the suspension was subjected to 10 min of irradiation by a 455 nm LED before the following UV-vis examination. The spectrum for subtraction was the mixture of neutral Cd–SNDI and Et_3N without irradiation.

For Figs. 3d and 8d, please refer to Materials and measurements, Manuscript for the experimental procedures of electrochemical impedance spectroscopy (EIS) examinations.

For Fig. 3e, the EPR sample of radical anionic Cd–SNDI was prepared from solid Cd–SNDI (*ca*. 2.6 mg, containing *ca*. 0.0025 mmol NDI moieties) as described in the caption of Supplementary Fig. 8. Then, 25 μL stock solution (0.1 M in DMF) of **1a** (*ca*. 0.0025 mmol) was added, and this sample was subjected to 10 min of irradiation by a 455 nm LED before the following EPR examination.

For Fig. 3f, please refer to Supplementary Experimental Methods of Transient Photocurrent Responses, Supplementary Information for experimental details.

For Figs. 4c and 4d, the reaction conditions were similar to those of Fig. 6, **1a**, **1b**, and **1c**. The dose of the photocatalyst was 0.0025 mmol, 0.05 equiv. for **1c**, **1b**, or 0.005 mmol, 0.1 equiv. for **1a**. The dose of electron donor was 3.60 mmol, 72 equiv.; The reaction time for **1c**, **1b**, and **1a** was 1 hour, 2 hours, and 4 hours, respectively.

For Fig. 4e, please refer to Typical Procedure for the Photocleavage of Aryl Halides, Supplementary Information for experimental details.

For Fig. 4f, the reaction conditions were similar to those of Table 1, entry 6, except for the additional employment of TEMPO (1.2 equiv.; Table 1, entry 15) as the radical scavenger. After the reaction, the reaction mixture was filtrated via a 0.22 μm filter, and the resulting clear solution was evaporated, and then devoted to HRMS for determining the TEMPO trapping product of aryl radical.

For Fig. 6, **1** to **13**, the photocatalyst was Cd–SNDI (0.0025 mmol, 0.05 equiv.), and the dose of electron donor dibutylamine was 3.60 mmol, 72 equiv.; For **14** to **24**, the photocatalyst was Cd–SNDI (0.005 mmol, 0.05 equiv.), and the dose of electron donor dibutylamine was 8 equiv.; Meanwhile, the amount of the trapping agent was 25 equiv.; For **14** to **22**, the solvent was 1 mL DMSO; For **23** and **24**, 3 mL solvent was used.

For Fig. 7b, the preparation methods of radical anionic Mg–NDI, Sr–NDI, and Cd–NDI were similar to that described in Supplementary Fig. 13.

Supplementary Table 1. Comparison of our work with the state-of-the-art in the photoreductive cleavage of aryl halides.

Photocatalyst / Light source	Maximum E_{red} (V *vs.* SCE)	Coupling reaction types	Advances to the pioneering work
PDI / 455 nm[1]	-1.87[2]	C-C	König's pioneering work on conPET
PTH / 380 nm[3]	-2.1~ -2.57	C-C	Enhanced reduction capability and workable in the air via a one-photon mechanism
NpMI / blue LED[4]	-3.3	C-C, C-P	Electron-generated radical anion was further irradiated to obtain extreme reduction potential at the mild condition
4-DPAIPN / 405 nm[5]	-3.4	C-C, C-P, C-B	Carbon dioxide radical anion generated *in situ* reduced photocatalyst to get extreme reduction potential
3CzEPAIPN / 456 nm[6]	-2.94	C-C, C-P, C-B	Photocatalyst family of donor-acceptor cyanoarenes can undergo the conPET process for extreme reduction potential
Cd–SNDI / 455 nm	-2.11	C-C, C-P, C-B, C-S	**This work**: dye assembly can promote the kinetics between the short-lived radical anion excited state and substrate

Supplementary Demonstration of Dye-Aggregation Improved PET for Inert Bond Cleavage

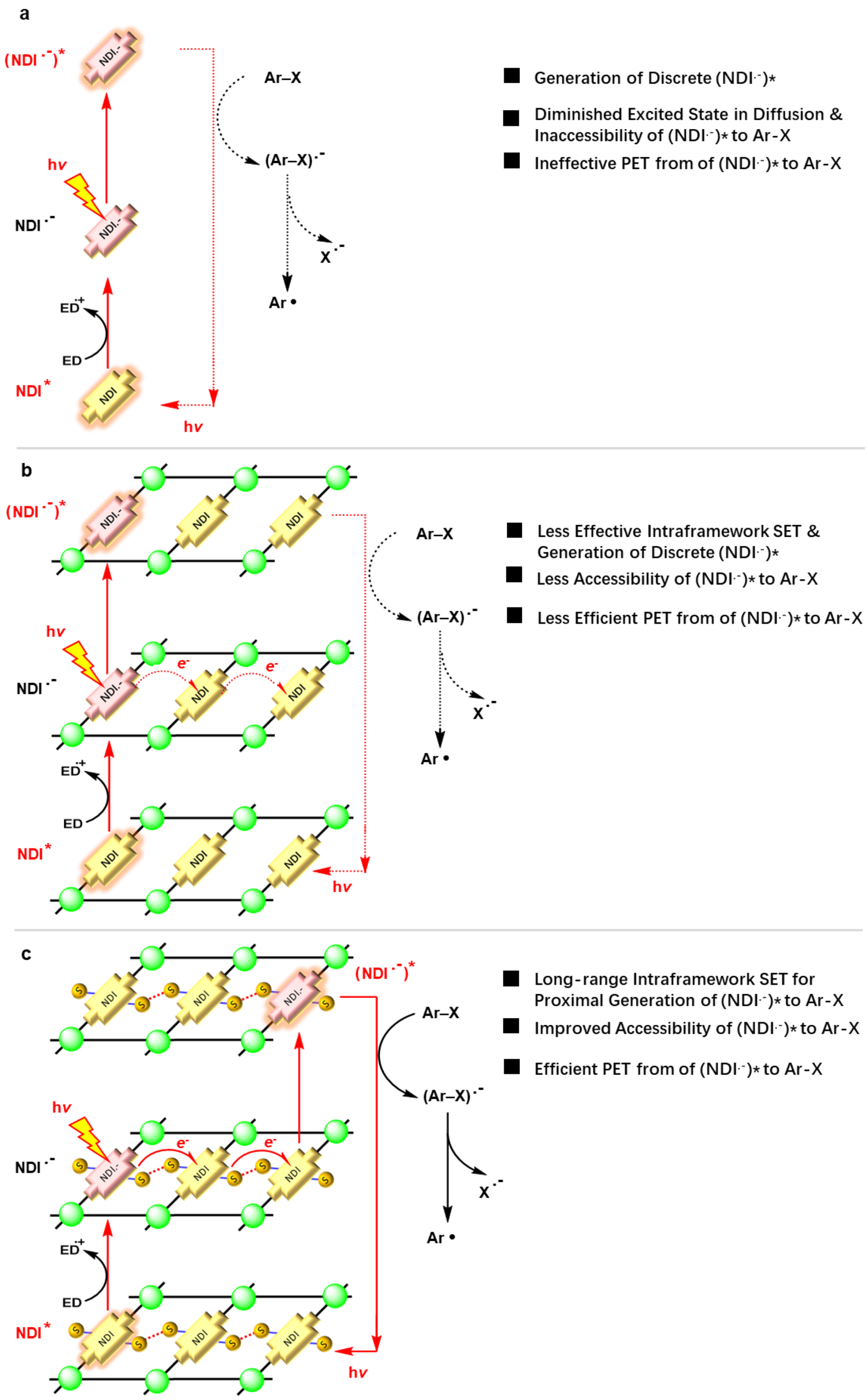

Supplementary Figure 1. Schematic demonstration of the specific dye-stacking model to improved PET for inert bond cleavage. Comparison of photoreductive cleavage of inert C_{Ar}–X bond catalysed by (**a**) homogeneous dye NDI in solution phase, (**b**) heterogeneous discretely arranged NDI in coordination polymer, and (**c**) heterogeneous chalcogen-bridged NDI string in coordination polymer, respectively. The “glow” with yellow or wine-red colours indicated the excited-state neutral NDI or radical anionic $NDI^{\bullet-}$.

Syntheses of NDI-based Coordination Polymers

Synthesis of H_4SNDI:

The compounds Br-NDA and H_4SNDI were synthesised according to the classical procedures[7]. Br-NDA (1.06 g, 2.49 mmol) was dissolved in 25 mL CH_3COOH within a 100.0 mL round-bottom flask. The mixture was heated at 60 ºC for 10 min before adding 5-amino-*m*-phthalic acid (905 mg, 5.00 mmol). After twelve hours of reflux at 120 ºC, 20 mL of deionised water was added to the resulting mixture at room temperature. Light pink Br-NDI was obtained in a yield of 83% (1.55 g) after filtration and drying. Br-NDI (1.55 g, 2.06 mmol) was dissolved in 10 mL dimethyl sulfoxide (DMSO) before removing oxygen from the reaction setup via N_2 bubbling for 15 min. NaSEt (520 mg, 6.19 mmol) was added, and the mixture was heated at 70 ºC in darkness for 24 h before cooling to room temperature. 100 mL deionised water was added to the reaction mixture, and H_4SNDI was precipitated by adjusting pH to 2.0~3.0 with HCl in darkness. After recrystallisation in acetic acid under the dark, H_4SNDI was obtained as a deep-red powder with a yield of 85% (1.26 g).

^{1}H-NMR (600 MHz, DMSO-*d*$_6$): δ 13.21 (br s, 4H; -CO_2**H**), 10.57 (s, 2H; naphthalene-**H$_2$**), 8.48 (s, 4H; 4,6-C$_{Ar}$-**H$_2$** of -Ph($CO_2H)_2$), 8.16 (s, 2H; 2-C$_{Ar}$-**H** of -Ph($CO_2H)_2$), 3.75 (br s, 2H; -SC**H$_2$**CH_3), 3.47-3.44 (m, 2H; -SC**H$_2$**CH_3), 1.22 (br s, 3H; -SCH_2C**H$_3$**), 1.08-1.06 (m, 3H; -SCH_2C**H$_3$**).

^{13}C-NMR (151 MHz, DMSO-*d*$_6$): δ 166.5 (-**C**O_2H), 140.0 (**C**$_{Ar}$), 131.7 (**C**$_{Ar}$), 124.4 (**C**$_{Ar}$), 123.4 (**C**$_{Ar}$), 56.0 (-S**C**H_2CH_3), 18.6 (-SCH_2**C**H_3).

If the procedures of -SEt decoration, workup, recrystallisation, and NMR sampling were done under ambient daylight, both SEt groups would be invisible in ^{1}H- and ^{13}C-NMR spectra, implying the possible presence of paramagnetic radical characteristics. Those results reflected that the photoirradiation from ambient daylight possibly enhanced the partial electron transfer from the SEt branch to NDI core of the H_4SNDI. After the esterification of H_4SNDI with MeOH and the successive HOAc recrystallisation in the presence of daylight, both SEt groups appeared again in ^{1}H-NMR. Inspired by this clue and the relative literature results[8-10], it was possible that the carboxylic acid groups played a role in enhancing the photosensitivity of intra-ligand charge transfer of H_4SNDI towards weak irradiation such as daylight.

HRMS (MALDI-TOF) m/z calcd. for $C_{34}H_{22}N_2O_{12}S_2^{+\cdot}$ $[M]^{+\cdot}$ 714.0614, found 714.0625.

Element analysis: Anal. Calcd (%) for $C_{38}H_{30}N_2O_{16}S_2$: C, 54.68; H, 3.62; N, 3.36. Found: C, 54.52; H, 3.59; N, 3.32.

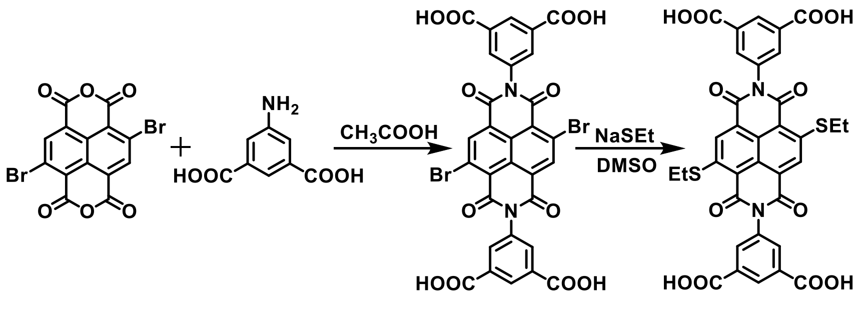

Synthetic steps of ligand H_4SNDI

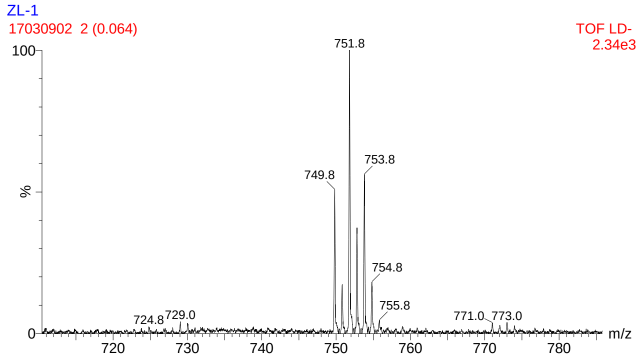

MALDI-TOF spectrum of Br-NDI (calculated value 749.88)

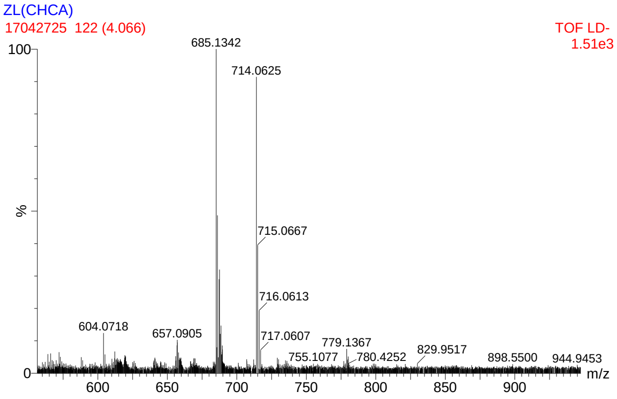

MALDI-TOF spectrum of H_4SNDI (calculated value 714.0614)

Synthesis of Cd–SNDI: H_4SNDI (14 mg, 0.020 mmol), $Cd(NO_3)_2{\cdot}4H_2O$ (12 mg, 0.040 mmol), 0.05 mL HCl (3 M) were dissolved in a mixed solution of 2.0 mL dimethylformamide (DMF) and 0.2 mL H_2O. The resulting mixture was heated in a 25 mL Teflon-lined autoclave at 100 °C for four days, then allowed to cool slowly to room temperature. Red to black crystals were obtained in 26% yield (based on H_4SNDI). Anal. Calcd (%) for Cd–SNDI ($CdC_{43.5}N_5S_2O_{15.5}H_{39}$): C, 49.46; H, 3.72; N, 6.63. Found: C, 48.80; H, 4.28; N, 7.09.

Synthesis of Cd–NDI: A mixture of H_4NDI (24 mg, 0.040 mmol), $Cd(NO_3)_2{\cdot}4H_2O$ (12 mg, 0.040 mmol), 0.08 mL HCl (3 M) were dissolved in 2.0 mL DMF. The resulting mixture was heated in a 25 mL Teflon-lined autoclave at 100 °C for three days, then allowed to cool slowly to room temperature. Orange to red crystals were obtained and suitable for X-ray structural analysis. Yield: 70% (based on H_4NDI). Anal. Calcd (%) for Cd–NDI ($C_{77}H_{68}CdN_{11}O_{28.5}$): C, 53.90; H, 3.99; N, 8.98. Found: C, 53.78; H, 4.07; N, 9.14.

Synthesis of pyrene@Sr–NDI: H_4NDI (21 mg, 0.035 mmol), $Sr(NO_3)_2{\cdot}4H_2O$ (24 mg, 0.085 mmol), pyrene (42 mg, 0.21 mmol), 0.2 mL HCl (3 M) were dissolved in 4.0 mL DMF. The resulting mixture was heated in a 25 mL Teflon-lined autoclave at 90 °C for 24 h, then allowed to cool slowly to room temperature. Red to black crystals were obtained in 70% yield (based on H_4NDI).

Supplementary Table 2. Crystallographic data for Cd–SNDI, Cd–NDI, and pyrene@Sr–NDI

Compound	Cd–SNDI	Cd–NDI	pyrene@Sr–NDI
Empirical formula	$CdC_{43.5}N_5S_2O_{15.5}H_{39}$	$C_{77}H_{68}CdN_{11}O_{28.5}$	$C_{3.41}H_{2.82}N_{0.35}O_{0.94}Sr_{0.12}$
Formula wt	1056.31	1715.82	74.13
T/K	200	293	150
Crystal system	Tetragonal	Monoclinic	Tetragonal
Space group	*P*-42c	*C*2/c	*I*41/a
a/Å	10.0891(7)	22.9797(9)	28.3250(11)
b/Å	10.0891(7)	22.7946(11)	28.3250(11)
c/Å	35.288(2)	18.5428(8)	13.7766(5)
β/deg	90.00	122.7830(10)	90.00
V/Å^3	3592.0(6)	8165.9(6)	11053.0(9)
Z	2	4	136
D_{calcd}/g	0.977	1.396	1.515
μ/mm^{-1}	0.410	0.355	2.007
No. of rflns collected	20696	7073	4864
No. of unique rflns	3106	6131	4500
R(int)	0.0459	0.0338	0.0568
F(000)	1078.0	3532.0	5136
R1 (I>2σ)^a	0.1051	0.0530	0.0262
R1 (all data)^a	0.1421	0.0614	0.0319
wR2 (I>2σ)^b	0.2750	0.1456	0.0611
wR2 (all data)^b	0.2931	0.1519	0.0627
GOF	0.956	1.054	1.079
CCDC number^c	2246332	1938743	2076326

$^a R1 = \sum||F_o| - |F_c||/\sum|F_o|$. $^b wR2 = |\sum w(|F_o|^2 - |F_c|^2)|/\sum|w(F_o^2)^2|^{1/2}$.

^{c}After refining cif file of Cd–SNDI to improve the quality of data analysis, the residual Level-B Alerts were listed and explained as below:

Alert level B:

(1) PLAT430_ALERT_2_B Short Inter D...A Contact O1 ..O301 . 2.63 Ang. y,1-x,-z = 3_565 Check

(2) PLAT430_ALERT_2_B Short Inter D...A Contact O1 ..N301 . 2.75 Ang. y,1-x,-z = 3_565 Check

(3) PLAT936_ALERT_2_B The Embedded .res File Includes a DAMP Command. 500.0 Report

Explanation to Alert (1) & (2): Atom O1 belongs to the carboxylate of Cd-SNDI, O301 and N301 belong to the lattice solvent DMF with partial occupation and structural disorder. These two alerts might be caused by spatial proximity of solvent DMF to the framework.

Explanation to Alert (3): The DAMP Command was used to stabilize the refinement.

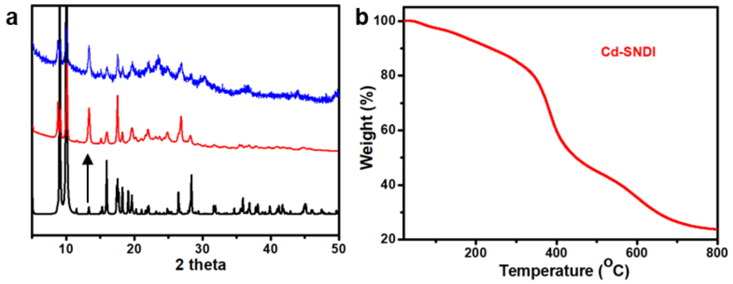

Supplementary Figure 2. (**a**) Comparative XRD patterns of the simulated (Black), freshly prepared (red), and recovered Cd–SNDI after impedance experiment (blue). The intensified peak owing to preferred facet orientation was highlighted by the arrow. (**b**) Thermogravimetric analysis (TGA) curve of Cd–SNDI.

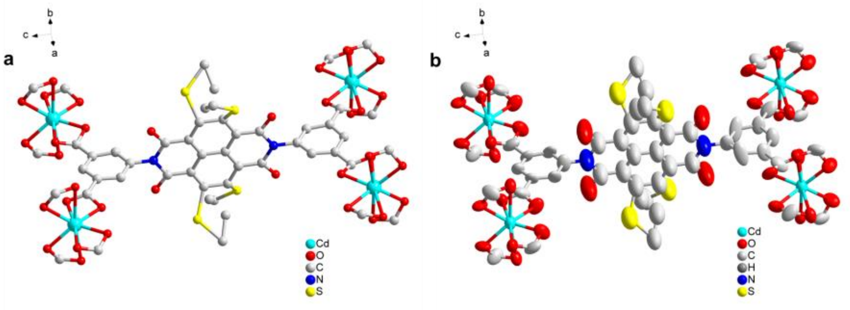

Supplementary Figure 3. (**a**) Ball-and-stick and (**b**) ellipsoid representation of the coordination environment Cd^{2+} and NDI unit within Cd–SNDI. For (**b**), the drawing was exhibited at 50% ellipsoid probability. The atoms of Cd, O, N, C, and S were drawn in cyan, red, blue, grey, and yellow, respectively. The solvent molecules and hydrogen atoms were omitted for clarity. The SEt groups were disordered in crystallography. Thus, it was drawn on every possible position.

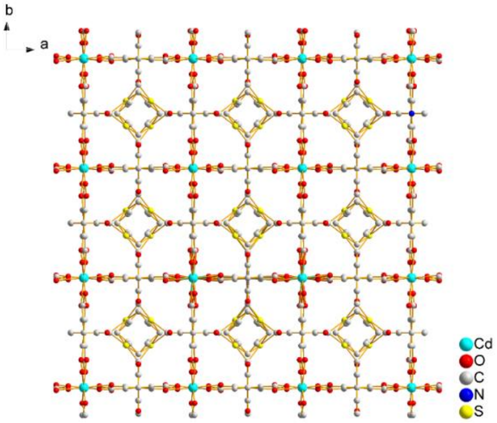

Supplementary Figure 4. Ball-and-stick representation of Cd–SNDI viewing from the *z*-axis. The setup was identical to Supplementary Figure 3.

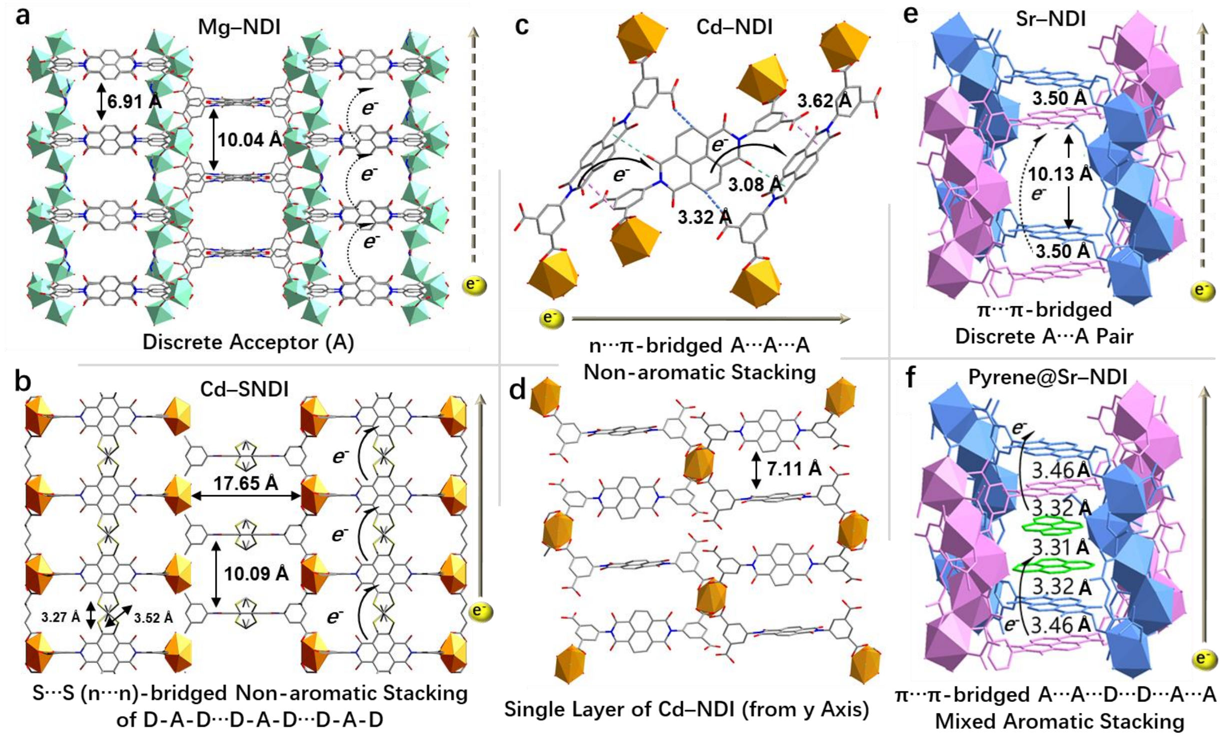

Supplementary Figure 5. Crystal structures showing the different arranging modes of NDI units in coordination polymers and the corresponding electron-delivering abilities. (**a**) The discrete NDI array results in poor electron communication within Mg–NDI. (**b**) S···S contact facilitates the electron transfer among the discrete NDI array in Cd–SNDI. (**c**) Sufficient n···π inter-ligand interactions render the interpenetrated Cd–NDI good electron-delivering ability. (**d**) The single layer of Cd–NDI shows the discrete arrangement of NDI. (**e**) The orthogonal stacking between adjacent NDI cores and the spatial isolation of neighboring NDI pairs hampered the inter-ligand electron transfer in Sr–NDI. (**f**) The encapsulation of two pyrene units into Sr–NDI leads to strong charge-transfer interaction and good electron-delivering ability for pyrene@Sr–NDI. The numbers in the crystal structures refer to the distances shown in angstroms. Colour code: light green, Mg-carboxylate cluster; light gold, Cd-carboxylate cluster; red, O; blue, N; light grey, C; light rose and light navy, two sets of Sr-carboxylate cluster, respectively; rose and navy, two sets of NDI ligands in Sr–NDI, respectively; lime, pyrene moieties. Electron acceptor, A; electron donor, D. The light gold arrows attached to coordination polymers illustrated the correlation between charge-transfer abilities and differentiated NDI stacking modes.

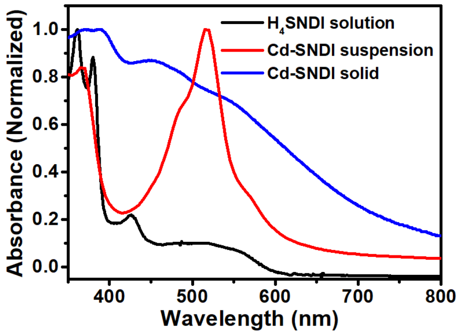

Supplementary Figure 6. The normalised UV-vis absorptions of the ligand H_4SNDI and coordination polymer Cd–SNDI. H_4SNDI solution was prepared by adding 1 mg H_4SNDI to 3 mL DMF. Cd–SNDI suspension was prepared by adding 2 mg Cd–SNDI into 3 mL DMF and the subsequent sonication. Cd–SNDI solid spectra was prepared by the compression of 10 mg mixed powder of Cd–SNDI/KBr (1:10). The spectra were normalised by setting the peak summit as 1.00.

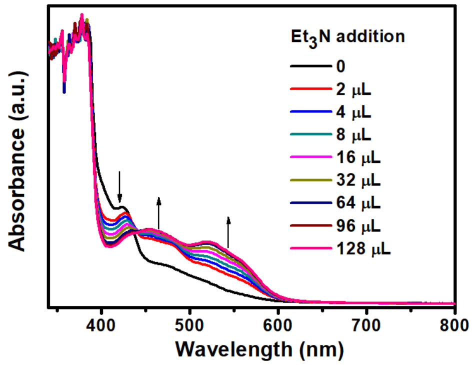

Supplementary Figure 7. Absorption changes of the ligand H_4SNDI upon adding the stock solution of Et_3N in DMF. The noted amount of Et_3N referred to the stock solution. 1 mg H_4SNDI was dissolved in 3 mL DMF, and the stock solution of Et_3N was prepared by adding 10 μL pure Et_3N to 200 μL DMF.

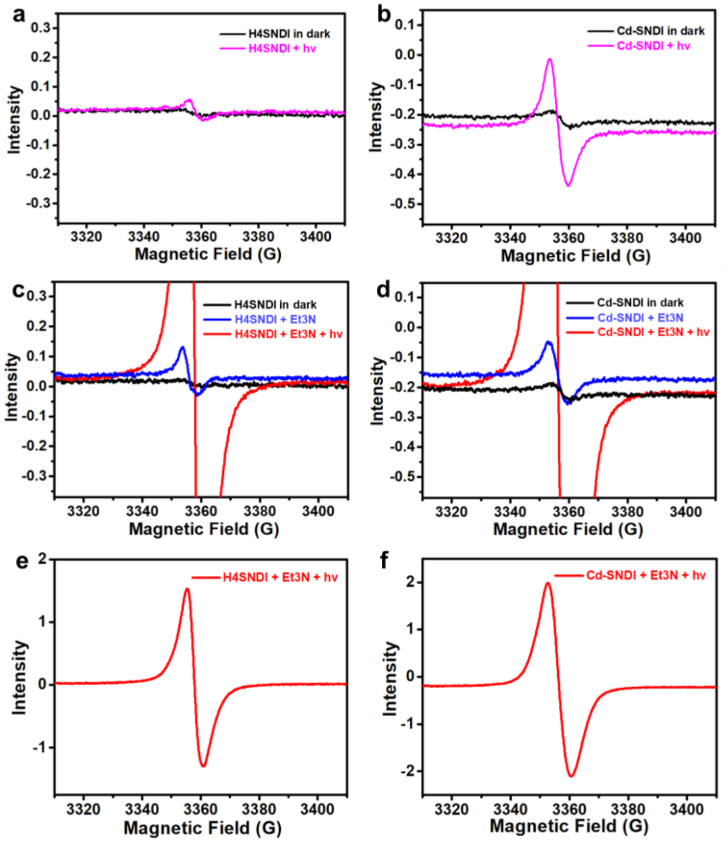

Supplementary Figure 8. Electron paramagnetic resonance (EPR) spectra of the solid samples of ligand H_4SNDI (**a**) and coordination polymer Cd–SNDI (**b**) under dark (black) *vs.* upon photoirradiation from 455 nm LED for 5 min (rose). The comparative EPR spectra of ligand H_4SNDI (**c**) and coordination polymer Cd–SNDI (**d**) in dark (black), with added Et_3N (blue), and treated with Et_3N and successive photoirradiation from 455 nm LED for 5 min (red). The global views of EPR spectra of radical anionic H_4SNDI (**e**) and radical anionic Cd–SNDI (**f**) that prepared by the protocols of (**c**) and (**d**), respectively. In the comparative EPR experiments, solid samples of H_4SNDI (*ca*. 1.8 mg, 0.0025 mmol) and Cd–SNDI (*ca*. 2.6 mg, 0.0025 mmol) containing similar molar amounts of NDI moieties were employed to ensure comparability. The samples were mounted in the 0.5 mm capillaries for photo-permeability, and then sealed in EPR tubes under an inert N_2 atmosphere. The excess amounts of Et_3N (*ca*. 5 μL) were used to treat the samples of ligand and coordination polymer.

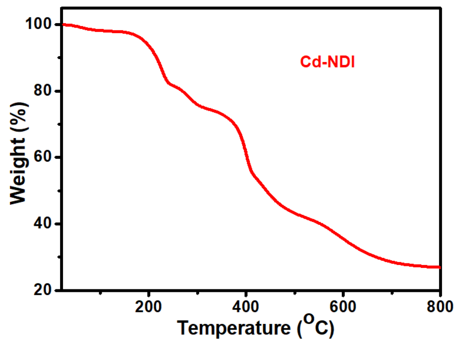

Supplementary Figure 9. TGA curve of Cd–NDI.

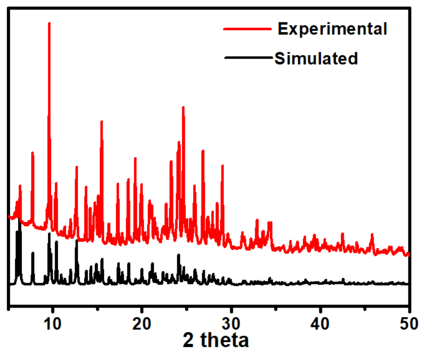

Supplementary Figure 10. The powder X-ray diffraction (PXRD) pattern of Cd–NDI.

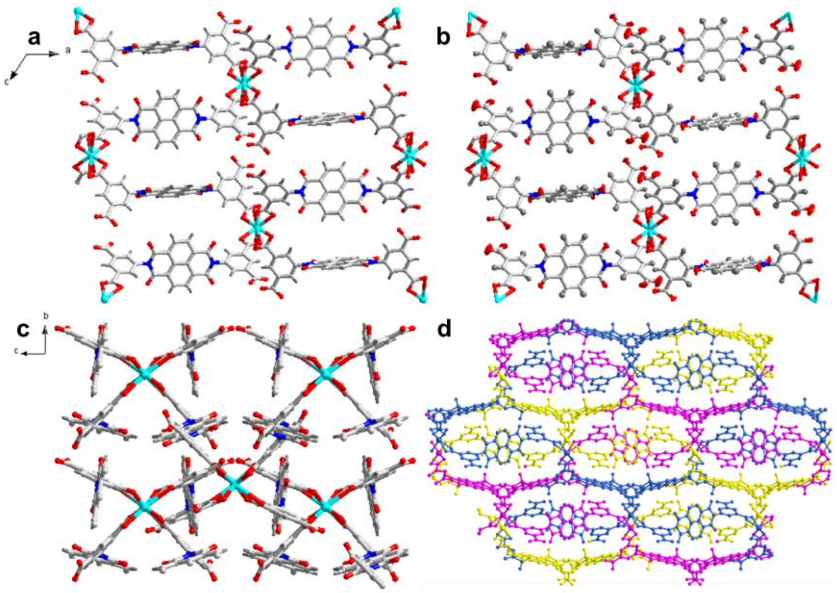

Supplementary Figure 11. (**a**) Ball-and-stick and (**b**) ellipsoid representations of one layer of Cd–NDI viewing from *y*-axis. For (**b**), the drawing was exhibited at 50% ellipsoid probability. (**c**) Ball-and-stick representation of interpenetrated Cd–NDI viewing from *x*-axis. The atoms of Cd, O, N, C, and H were drawn in cyan, red, blue, grey, and dark grey, respectively. The solvent molecules were omitted for clarity. (**d**) Ball-and-stick representation of the three-interpenetrated nets of Cd–NDI with each net in one colour.

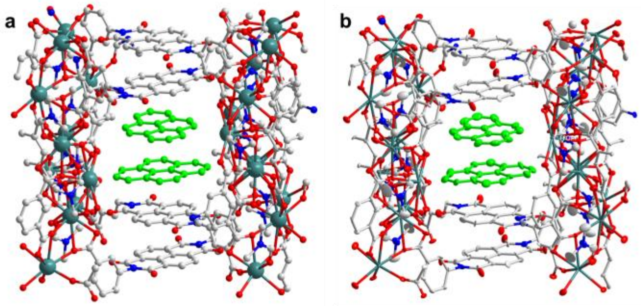

Supplementary Figure 12. (**a**) Ball-and-stick and (**b**) ellipsoid representations of pyrene@Sr–NDI. Colour code: teal green, Sr; red, O; blue, N; light grey, C of Sr–NDI framework; lime, C of pyrene guest molecules.

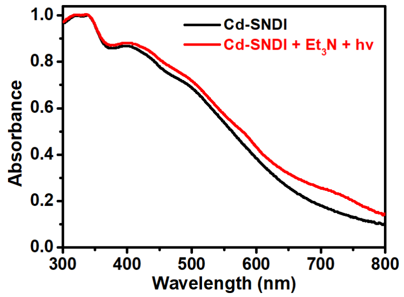

Supplementary Figure 13. The normalised solid-state UV-vis absorption of coordination polymer Cd–SNDI and the radical anionic Cd–SNDI obtained by adding Et_3N and shining light (455nm LED). Cd–SNDI solid sample was prepared by the compression of 10 mg mixed powder of Cd–SNDI/KBr (1:10), and the generation of radical anionic Cd–SNDI was similar to Fig. 3b except for adding Et_3N and shining light towards the solid sample.

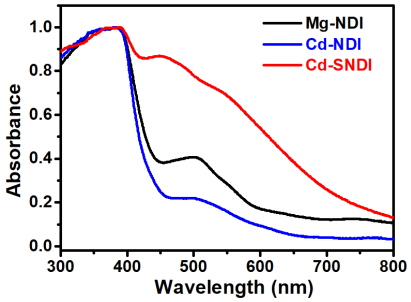

Supplementary Figure 14. The normalised solid-state UV-vis absorption of Mg–NDI, Cd–NDI, and Cd–SNDI.

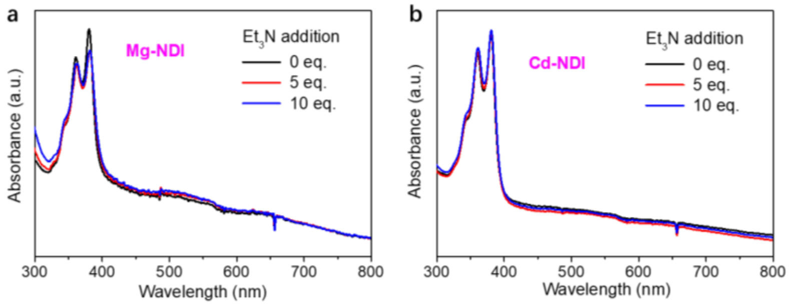

Supplementary Figure 15. The normalised absorptions of the DMF suspension of Mg–NDI (**a**) and Cd–NDI (**b**) upon Et_3N addition. The preparations of the coordination polymer suspensions were similar to that of Fig. 3b except for using Mg–NDI or Cd–NDI.

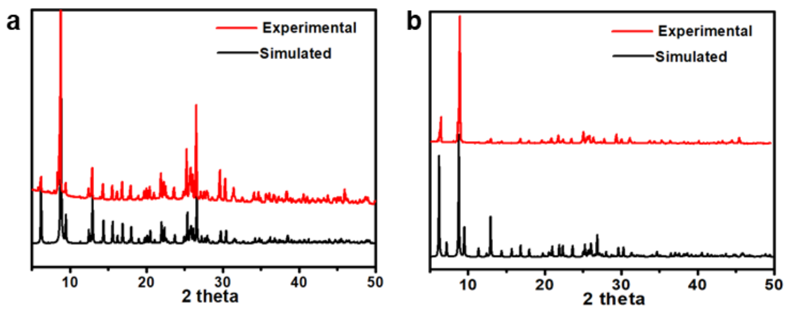

Supplementary Figure 16. PXRD pattern of Sr–NDI (**a**) and pyrene@Sr–NDI (**b**).

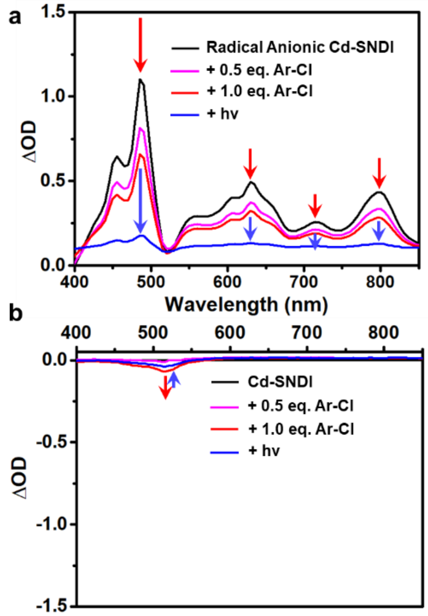

Supplementary Figure 17. Comparative study on the difference absorption spectra of (**a**) radical anionic Cd–SNDI and (**b**) neutral Cd–SNDI with titration of **1a**, the typical Ar-Cl substrate (red arrows), followed by 455 nm LED photoirradiation (blue arrows). In the case of (**a**), the radical anionic Cd–SNDI was obtained by treating the DMF suspension of Cd–SNDI with the addition of Et_3N and successive 455 nm LED photoirradiation. The experimental details of (**a**) were the same as that of Fig. 3c, as shown in Experimental Details for Figures in Manuscript. For (**a**), the spectrum for subtraction was the mixture of neutral Cd–SNDI and Et_3N without irradiation. The experimental details of (**b**) were similar to that of Fig. 3c, except for the absence of generating radical anionic Cd–SNDI. For (**b**), the spectrum for subtraction was the DMF suspension of neutral Cd–SNDI.

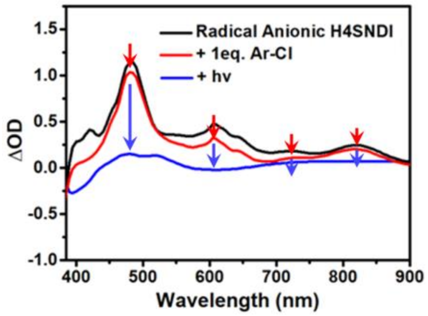

Supplementary Figure 18. The difference absorption spectra of radical anionic H_4SNDI solution with the addition of a similar amount of **1a**, the typical Ar-Cl substrate (red arrows), followed by 455 nm LED photoirradiation (blue arrows). The radical anionic H_4SNDI was obtained by treating the DMF solution of neutral H_4SNDI with the addition of Et_3N and successive 455 nm LED photoirradiation. The experimental details were similar to that of Fig. 3c except for using H_4SNDI stock solution (3.6 mg H_4SNDI in 2 mL DMF) instead of coordination polymer suspension. The spectrum for subtraction was the mixture of neutral ligand and Et_3N without irradiation.

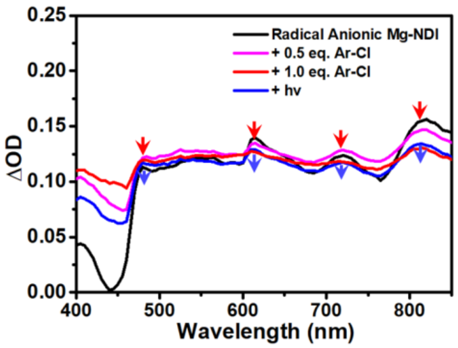

Supplementary Figure 19. The difference absorption spectra of radical anionic Mg–NDI with the addition of similar amount of **1a**, the typical Ar–Cl substrate (red arrows), followed by 455 nm LED photoirradiation (blue arrows). The radical anionic Mg–NDI was obtained by treating the DMF suspension of neutral Mg–NDI with addition of Et_3N and successive 455 nm LED photoirradiation. The experimental details were similar to that of Fig. 3c except for using Mg–NDI (*ca*. 3.8 mg) instead of Cd–SNDI. The spectrum for subtraction was the mixture of neutral Mg–NDI and Et_3N without irradiation.

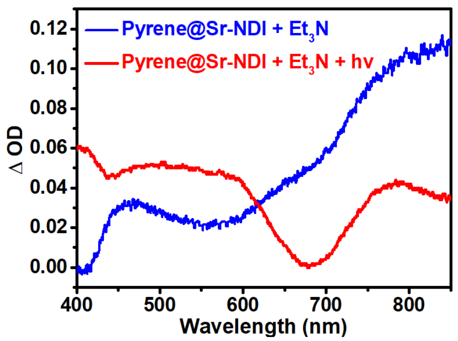

Supplementary Figure 20. Difference absorption spectra of Pyrene@Sr–NDI with Et_3N addition (blue line) followed by irradiation (red line). The preparation of the solid sample, the addition of Et_3N, and successive photoirradiation were handled similarly to Supplementary Fig. 13. The spectrum for subtraction was the neutral Pyrene@Sr–NDI.

Supplementary Table 3. Estimation of the reducing potential of excited-state radical anionic NDI-based coordination polymer/ligand.

Coordination Polymer/Ligand	E^0(NDI$^{\bullet-}$/NDI) (V *vs* SCE)	$D_0 \rightarrow D_1$ Transition (nm)	E^0(*NDI$^{\bullet-}$/NDI) (V *vs* SCE)[a]
Cd–SNDI	-0.57	800	-2.12 V
H_4SNDI	-0.54	788	-2.11 V
Cd–NDI	-0.54	780	-2.13 V
Mg–NDI	-0.55	735	-2.24 V
Sr–NDI	-0.50	780	-2.09 V
pyrene@Sr–NDI	-0.46	785	-2.04 V

[a] E^0(*NDI$^{\bullet-}$/NDI) = E^0(NDI$^{\bullet-}$/NDI) - $E^{0\text{-}0}$(*NDI$^{\bullet-}$), and $E^{0\text{-}0}$(*NDI$^{\bullet-}$) was roughly estimated by $D_0 \rightarrow D_1$ transition according to literatures[1,2].

Supplementary Experimental Methods and Analyses of Transient Absorption Spectra

Details of the femtosecond pump-probe TA measurements were similar to the literature procedures[11]. Briefly, the laser source was a regenerative amplified Ti:sapphire laser system (Coherent; 800 nm, 70 fs, 6 mJ/pulse, 1 kHz repetition rate). The 800 nm output pulse was split into two parts with a 50% beam splitter. One part was used to pump an OPA, which can generate a wavelength-tunable laser pulse from 250 nm to 2.5 μm using as a pump beam. Another part was attenuated with a neutral density filter and focused into a sapphire or CaF_2 crystal to generate a white light continuum for the probe beam. The pump pulses were chopped by a synchronised chopper at 500 Hz, and the absorbance change was calculated with two adjacent probe pulses (pump-blocked and pump-unblocked). The delay between the pump and probe pulses was controlled by a motorised delay stage.

The samples of neutral ligand H_4SNDI and neutral coordination polymer Cd–SNDI were the corresponding DMF solution and the finely-grinded and dispersed suspension in DMF, respectively. For all fs TA measurements, samples were filled in 1 mm airtight cuvettes prepared in a N_2-filled glove box and measured under ambient conditions, and the UV-visible (UV-vis) absorbances of all samples were adjusted to 0.5 before further characterisation. The samples of radical anionic H_4SNDI and radical anionic Cd–SNDI were prepared by treating the corresponding DMF solution of neutral H_4SNDI or DMF suspension of neutral Cd–SNDI with excess amounts of degassed Et_3N (~2 μL) and successive photoirradiation from a 455 nm LED for 5 min, respectively.

Owing to the highly complex nature of fs-TA spectra and the tight correlations among the peaks, decay/recovery lifetime, and other information of various NDI samples measured at different excitation wavelengths. Herein, it was necessary to analyse those data comprehensively in a comparative manner, which was helpful to decrease the misleading based upon the fragmented information.

Considering the critical role of the peaks in the range of 350 to 500 nm for the assignment of excited-state species, we tested the fs-TA spectra of the radical anion samples of Cd–SNDI and H_4SNDI under the excitation of 630 nm (Fig. 5). This wavelength can excite the characteristic peaks of $NDI^{\bullet-}$ of radical anionic ligand or radical anionic coordination polymer, and also avoid the excitation of neutral species. After the 630 nm laser excitation of radical anionic samples for 1 ps, the excited state absorption (ESA) bands and the ground state bleach (GSB) bands could be clearly observed as black lines. For radical anionic Cd–SNDI (Fig. 5c), the GSB peak was observed at ~375 nm, and the ESA band covered a broad range from 400 nm to 550 nm, of which the decay lifetime (τ = 164 ps, Fig. 5d) was close to the reported data of $(NDI^{\bullet-})^*$ (such as τ = 142 ps)[12]. In comparison, in the case of radical anionic H_4SNDI (Fig. 5a), the GSB band was blue-shifted to less than 350 nm, and the ESA band was also blue-shifted and exhibited a narrower shape, of which the decay lifetime was shorter than that of radical anionic Cd–SNDI (τ = 93 ps, Fig. 5b), but still not far from some literature result (such as τ = 112 ps)[13]. For both cases, the possibly existing GSB bands of $NDI^{\bullet-}$ moieties at ~480 nm might be compensated by the intense ESA bands. From 80 ps after irradiation on radical anionic Cd–SNDI, a new bleach at ~425 nm appeared (Fig. 5c), which might be assigned to the vanished intra-ligand charge-transfer band (see the CT band of ligand at ~425 nm, Fig. 2a). In comparison, the bleaching of the intra-ligand charge-transfer band was not observed in the case of radical anionic H_4SNDI (Fig. 5a). The vanishment of the intra-ligand charge-transfer band within Cd–SNDI implied the possible tendency of inter-ligand electronic communication.

In comparison to the 630 nm wavelength, the 480 nm pump laser could irradiate the intra-ligand charge transfer band of neutral H_4SNDI (Fig. 2a) and the $D_0 \rightarrow D_n$ doublet-transition peak of $NDI^{\bullet-}$ (Figs. 3a, 3b), which was also close to the 455 nm wavelength of LED in the practical consecutive photon excitation.

After irradiating neutral H_4SNDI from 480 nm laser for ~5 ps (Supplementary Fig. 22a), the discrete or merged doublet-state peaks of $NDI^{\bullet-}$ could be detected at the locations of 480~500 nm, 630~650 nm, and 680~750 nm, and the positive band at 540~560 nm resembled the typical two-centre three-electron $[S\therefore\pi]^+$ radical cationic species[14-17]. The *in situ*-generated radical ionic pairs indicated the strong tendency of photoinduced charge separation of H_4SNDI, and the similar evidences were also revealed in the case of neutral Cd–SNDI (Supplementary Fig. 22b). Moreover, the curve sections that bleached at 520 nm (Supplementary Figs. 22a, 22b) were possibly assigned to the vanished intra-ligand charge transfer (see the CT band centred at ~520 nm, Fig. 2a), which might be contributed by the competition from inter-ligand electronic communications.

The fs-TA spectra of radical anionic Cd–SNDI and radical anionic H_4SNDI were also examined under the excitation of a 480 nm pump laser (Supplementary Fig. 21). ~5 ps after irradiation, an ESA band covering 500~600 nm centred at 560 nm was observed in the case of radical anionic H_4SNDI (Supplementary Fig. 21a), and the radical anionic Cd–SNDI exhibited several merged positive bands that covered a range from 550~750 nm (Supplementary Fig. 21c). The positive peaks at 540~560 nm resembling the typical two-centre three-electron $[S\therefore\pi]^+$ species were also found for both cases[14-17], implying the intra-ligand charge transfer between the SEt branch and aromatic NDI nucleus. It was noteworthy that this positive band centred at 560 nm decayed much faster in the case of radical anionic Cd–SNDI (Supplementary Fig. 21d) than that of radical anionic H_4SNDI (Supplementary Fig. 21b), further implying the possible presence of inter-ligand charge transfer in coordination polymer as a competitive route of the intra-ligand charge transfer. Moreover, the remarkable and long-lived bleaching peak at ~520 nm of radical anionic Cd–SNDI might be assigned to the vanished intra-ligand charge-transfer band (see the intra-ligand CT-band centred at ~520 nm, Fig. 2a), of which the ns-scaled recovery lifetime of 7.4 ns (Supplementary Fig. 21e) was comparable to the 8.2 ns decay lifetime of ~630 nm summit (Supplementary Fig. 21f). The correlation between this pair of GSB/ESA bands suggested that the ~630 nm positive peak with a long decay lifetime might be related to the inter-ligand charge transfer in the coordination polymer. In contrast, either the bleaching peak at ~520 nm or the positive one at ~630 nm was not found in the case of radical anionic H_4SNDI (Supplementary Fig. 21a), which did not support the profound inter-ligand charge transfer among radical anionic ligands in solution.

The apparent differences of fs-TA spectra between neutral (Supplementary Figs. 22a, 22b) and radical anion samples (Supplementary Figs. 21a, 21c) for both ligand H_4SNDI and coordination polymer Cd–SNDI excluded the remarkable presence of neutral NDI species in radical anionic samples of ligand and coordination polymer. Moreover, it was interesting to find the clues of inter-ligand charge transfer either in neutral or radical anionic Cd–SNDI, which was essential to understand the role of S⋯S contact in mediating inter-ligand electronic communications, and further interpret the structure-activity relationships regarding the enhanced accessibility of $(NDI^{\bullet-})^*$ towards Ar–X substrate in radical anionic Cd–SNDI.

It was reported that the excited-state aromatic diimide bis(radical anion)s $(ADI^{\bullet-})^*$-$(ADI^{\bullet-})$ could undergo inter-ADI charge transfer to form the dianionic NDI^{2-} and neutral NDI^0, demonstrating that the $(ADI^{\bullet-})^*$ functioned not only as electron donors but also as acceptors[13]. Moreover, it was known that the charge transfer also occurred between the closely associated $ADI^{\bullet-}$ and other redox-active counterparts, leading to the formation of NDI^{2-} under photoirradiation[18,19]. The following back-electron transfer between charge-separated pair involving the more stable close-shell dianionic NDI^{2-} could regenerate radical anionic $NDI^{\bullet-}$ with the semi-stable open-shell structure, and this process sometimes exhibited the ns-scale decay lifetime in fs-TA spectra[13].

Inspired by those literature information, the charge transfer between $(NDI^{\bullet-})^*$ and nearby electron-donating moieties such as SEt branches and $NDI^{\bullet-}$ could be expected in the case of radical anionic Cd–SNDI. Under 480 nm laser irradiation, the fs-TA curve characteristics of radical anionic Cd–SNDI and the corresponding decay/recovery lifetimes (Supplementary Figs. 21c, e, f) resembled the above-mentioned photoinduced charge transfer between $(NDI^{\bullet-})^*$ and neighbouring electron-donating counterpart to form dianionic (NDI^{2-})[18,20] and the following ns-scale back-electron transfer process involving dianionic (NDI^{2-}) species to regenerate $NDI^{\bullet-}$ [13]. Moreover, after the fast decay of intra-ligand charge transfer ESA at 560 nm in radical anionic Cd–SNDI (Supplementary Fig. 21d), the recovery of this ESA band after ~1 ns (with a recovery lifetime $\tau 3$ = 767 ps, Supplementary Fig. 21d) possibly suggested the regeneration of $NDI^{\bullet-}$ in coordination polymer.

It was well known that the close and continuous stacking of radical anionic aromatic diimide $ADI^{\bullet-}$ moieties in supramolecular assembly greatly enhanced the charge transfer along the stacking direction[21,22]. And the S···S contact was proved effective in mediating the remarkable electronic conductivity in coordination polymer[23]. Thus, the inter-ligand S···S contact was believed to facilitate charge transfer between neighbouring NDI moieties in Cd–SNDI, possibly allowing the position shifting of $(NDI^0)/(NDI^{\bullet-})/(NDI^{2-})$[24] along the NDI string.

Thus, based upon the pioneering works and the comparative fs-TA studies here, it was reasonable to deduce that the iterative rounds of photoinduced inter-ligand charge separation between neighbouring ligands, shifting of separated charges along NDI string, and the inter-ligand charge recombination in radical anionic Cd–SNDI might regenerate $NDI^{\bullet-}$ at remote locations of NDI string that near to the external substrate molecules (Figs. 1d to 1g)[25], which was believed to facilitate their encountering and reaction, circumvent the diffusion-limited PET between the extremely short-lived excited-state radical anion and substrate in solution phase, and alleviate the back-electron transfer from the one-electron reduced $[Ar–X]^{\bullet-}$ to the framework.

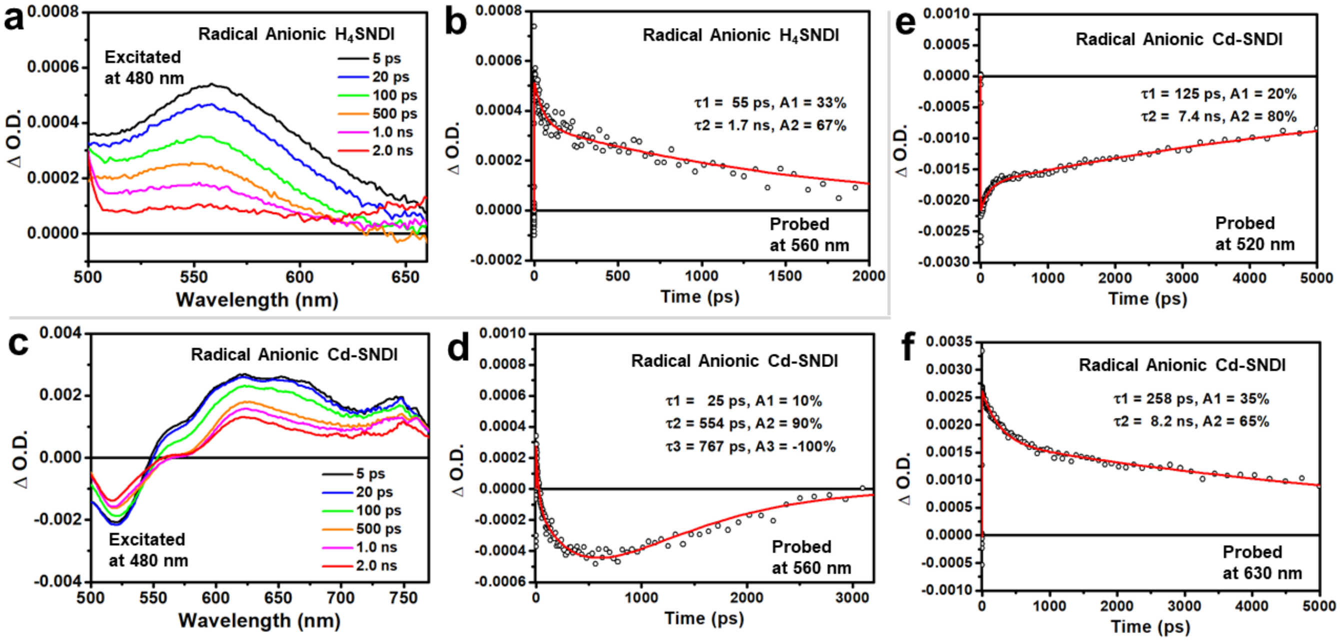

Supplementary Figure 21. The 480 nm-laser excited fs-TA spectra of (**a**) radical anionic ligand H_4SNDI and (**c**) radical anionic coordination polymer Cd–SNDI at the indicated delay/recovery times. And the corresponding kinetic traces of ΔO.D. probed at (**b**) 560 nm of radical anionic H_4SNDI and (**d**) 560 nm, (**e**) 520 nm, and (**f**) 630 nm of radical anionic Cd–SNDI, respectively.

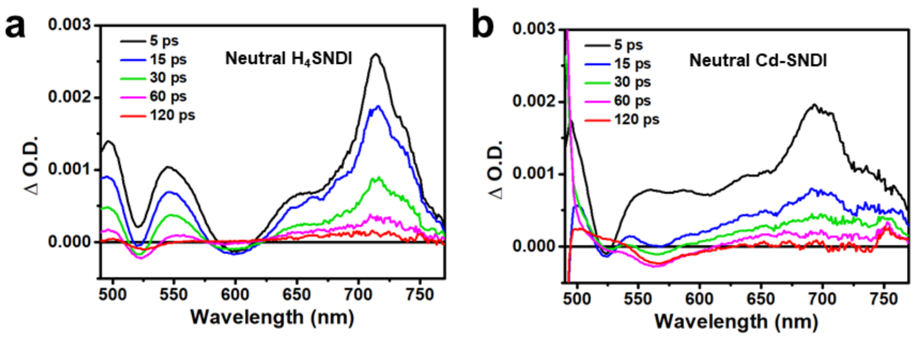

Supplementary Figure 22. The 480 nm-laser excited fs-TA spectra of (**a**) neutral ligand H_4SNDI and (**b**) neutral coordination polymer Cd–SNDI at the indicated delay/recovery times.

Supplementary Experimental Methods of Transient Photocurrent Responses

The photocurrent experiments were measured on ZAHNER ENNIUM Electrochemical Workstation with a typical three-electrode system. The sample mounted on ITO served as the working electrode. Taking Cd–SNDI as an example, 2 mg coordination polymer was well dispersed with 30 µL Nafion, 250 µL ethanol, and 250 µL H_2O, and then finely grinded. After mounting the sample onto ITO, the composite photocathode was oven dried. A platinum wire and Ag/AgCl electrode served as the counter and reference electrodes, respectively. The electrolyte was an NH_4PF_6 solution in CH_3CN (1.0 M), and the typical Ar–Cl substrate **1a** was used as the electron sink under the inert N_2 atmosphere (*ca*. 0.033 mmol/mL in CH_3CN). 455 nm LED was used as the light source. The transient photocurrent responses were taken with the photocathode biased at *ca*. 0.3 V, as shown in Fig. 3f.

Typical Procedure for the Photocleavage of Aryl Halides

A glass tube was filled with aryl halide (0.05 mmol, 1 equiv.), photocatalyst, mini-stirrer, and dry DMF (3 mL) as solvent. The resulting mixture was degassed with N_2 bubbling for 20 minutes, and then sealed. An electron donor was added during the bubbling process. The reaction mixture was irradiated at 40 ^{o}C by a homemade 455 nm LED loop. After the reaction, the reaction mixture was filtrated via a 0.22 µm filter, and the resulting clear solution was devoted to gas chromatography (GC) analysis. The yields were calculated from GC measurements using internal standards.

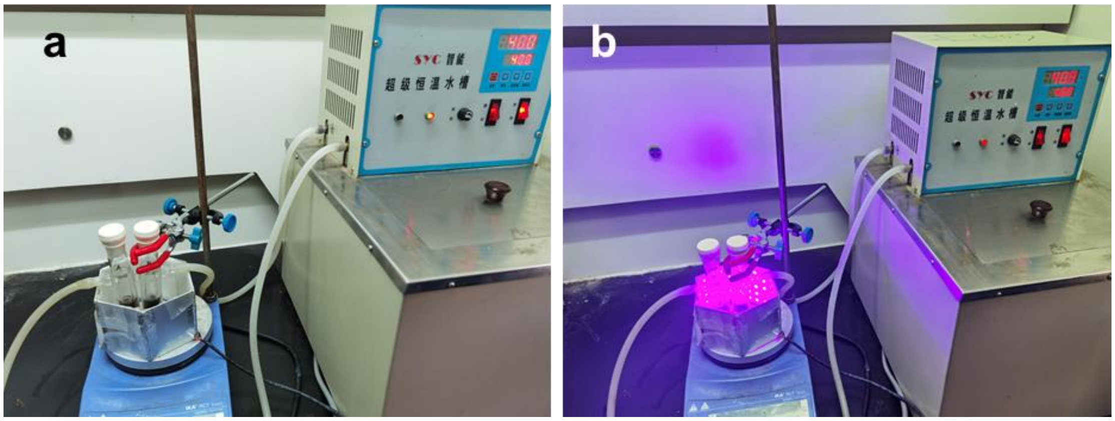

Supplementary Figure 23. Pictures of the setups for the photocleavage of aryl halides in the (**a**) absence or (**b**) presence of irradiation from 455 nm LED loop.

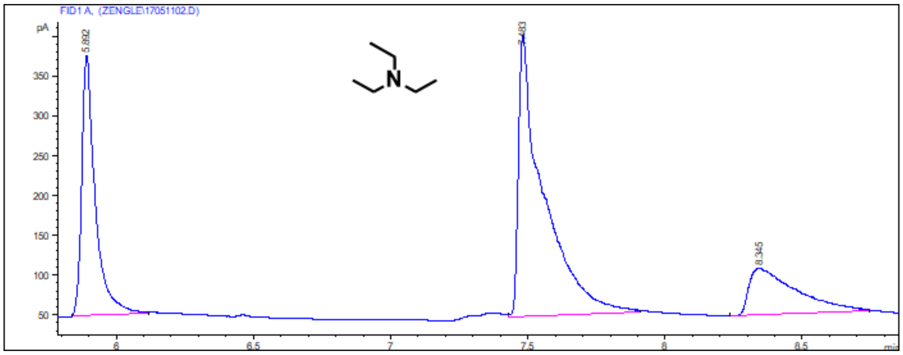

Supplementary Figure 24. GC spectra of the photoreduction of 4’-bromoacetophenone catalysed by Cd–SNDI in Fig. 4e. Product yield was calculated to be 71% using an internal standard. Triethylamine was used as electron donor.

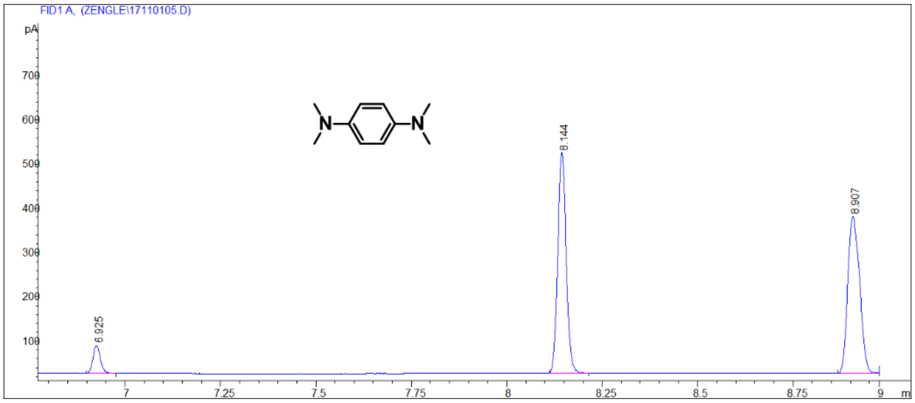

Supplementary Figure 25. GC spectra of the photoreduction of 4’-bromoacetophenone catalysed by Cd–SNDI in Fig. 4e with *N*,*N’*-tetra-methylphenylenediamine as electron donor (product yield 50%).

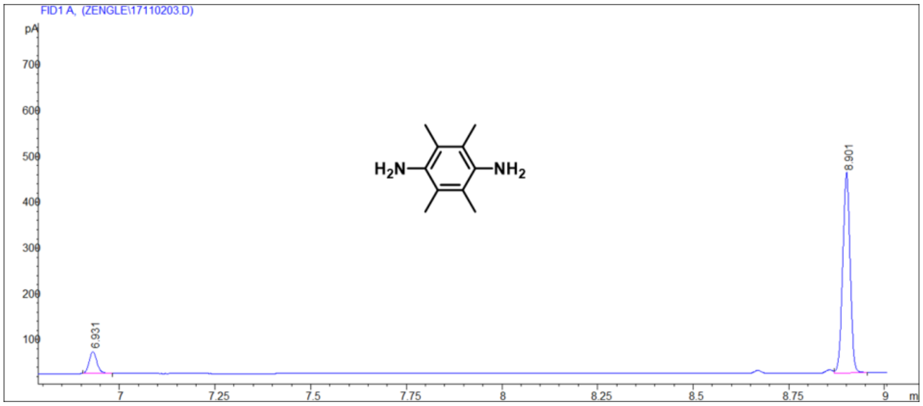

Supplementary Figure 26. GC spectra of the photoreduction of 4’-bromoacetophenone catalysed by Cd–SNDI in Fig. 4e with 2,3,5,6-tetramethylbenzene-1,4-diamine as electron donor (product yield 28%).

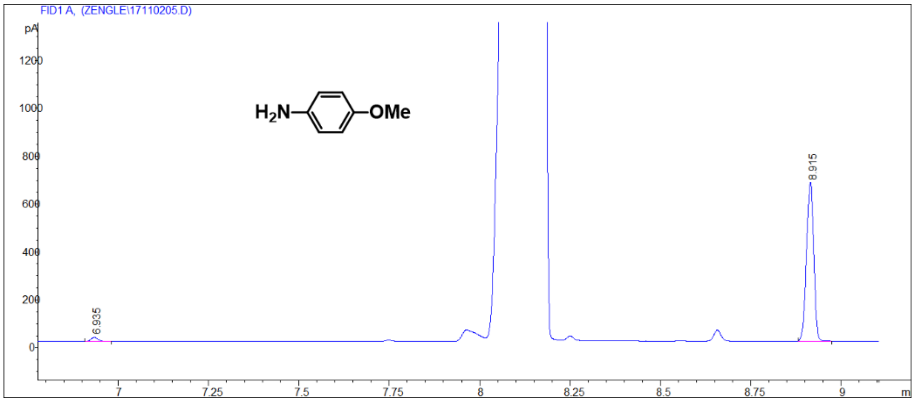

Supplementary Figure 27. GC spectra of the photoreduction of 4’-bromoacetophenone catalysed by Cd–SNDI in Fig. 4e with *p*-Anisidine as electron donor (product yield 8%).

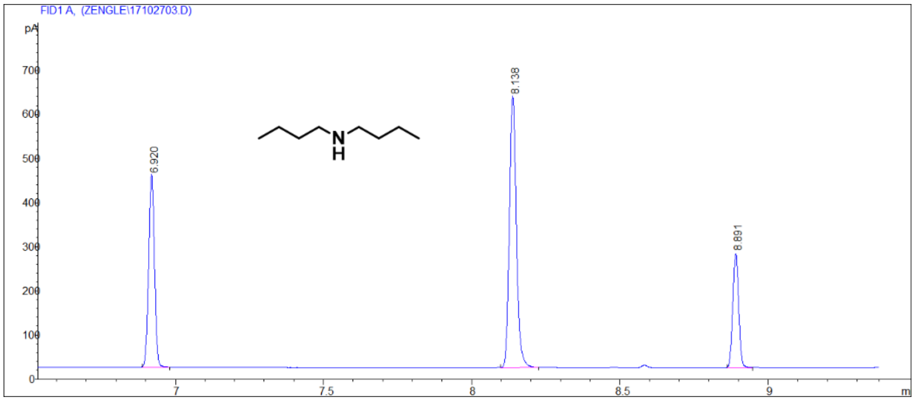

Supplementary Figure 28. GC spectra of the photoreduction of 4’-bromoacetophenone catalysed by Cd–SNDI in Fig. 4e using dibutylamine as electron donor (product yield 96%).

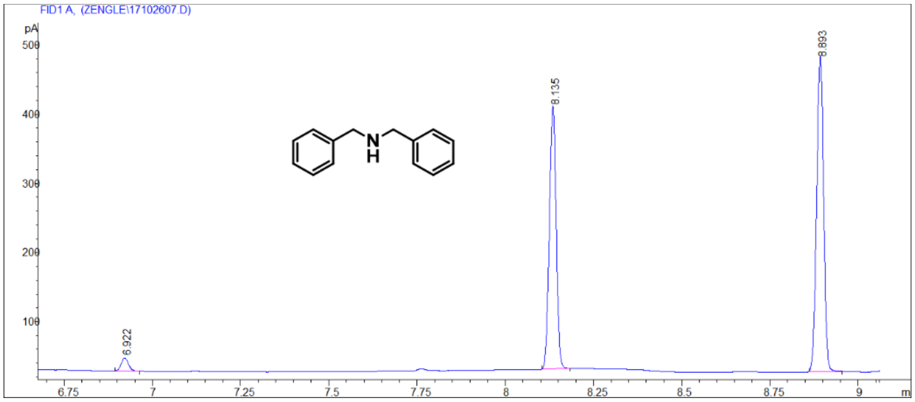

Supplementary Figure 29. GC spectra of the photoreduction of 4’-bromoacetophenone catalysed by Cd–SNDI in Fig. 4e using dibenzylamine as electron donor (product yield 46%).

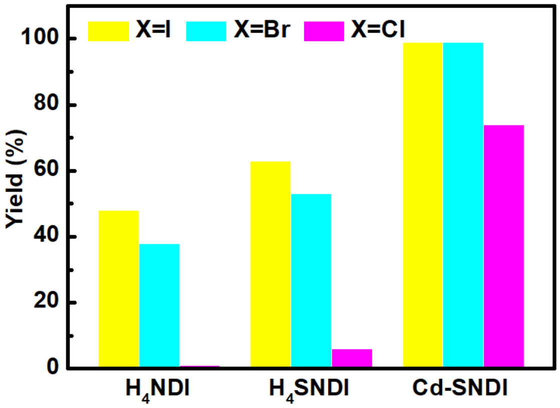

Supplementary Figure 30. Comparison of photocleavage results of aryl halides catalysed by ligand H_4NDI, H_4SNDI, and coordination polymer Cd–SNDI, respectively, using the optimal conditions of Fig. 6, **1a**, **1b**, **1c**, unless different kinds of photocatalysts.

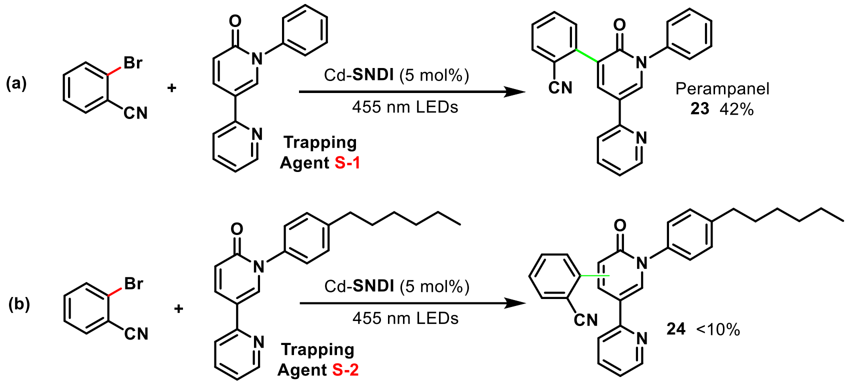

Supplementary Figure 31. Comparative study on photoreductive cleavage of C_{Ar}–Halide bond and radical coupling using the (**a**) normal-sized and (**b**) bigger-sized trapping reagents. Conditions: substrate 2-bromobenzonitrile **4b** (0.1 mmol, 1 equiv.), Cd–SNDI (0.05 equiv.), dibutylamine (8 equiv.), trapping agent (25 equiv.), DMSO (3 mL), 40 ºC, 455 nm LED, 4 h. When the bigger-sized trapping agent 1'-(4-hexylphenyl)-[2,3'-bipyridin]-6'(1'*H*)-one was used, the target product could not be purified from the unassignable regioisomers and side-products.

Ingress and Egress of Trapping Reagent within Coordination Polymer Cd−SNDI.

Crystals of coordination polymer Cd−SNDI (0.02 mmol) was soaked in a solution of either the normal-sized trapping agent 1'-phenyl-[2,3'-bipyridin]-6'(1'*H*)-one (0.10 mmol) or the bigger-sized trapping agent 1'-(4-hexylphenyl)-[2,3'-bipyridin]-6'(1'*H*)-one (0.10 mmol) in d^6-DMSO (0.5 mL), and the mixture was kept in an NMR tube and shaken by a vortex reactor. The uptake amount of the trapping agent within coordination polymer crystals was monitored by time-course ^{1}H-NMR of supernatant. The previously obtained coordination polymer crystals saturated with trapping agent were carefully wiped by the wet filter paper containing d^6-DMSO to remove the trapping agent absorbed on the surface of the crystals. Then, the crystals were immersed in d^6-DMSO (0.5 mL) and shaken by a vortex reactor. The amount of trapping agent released from coordination polymer was monitored by time-course ^{1}H-NMR of supernatant.

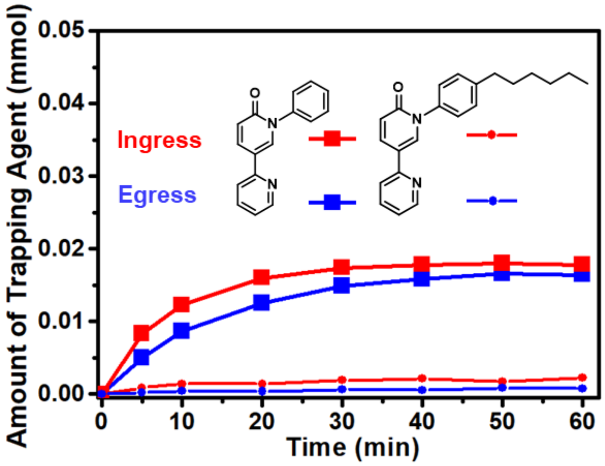

Supplementary Figure 32. Comparison of ingress (red line) and egress (blue line) of the normal-sized trapping agent 1'-phenyl-[2,3'-bipyridin]-6'(1'*H*)-one (square) and the bigger-sized trapping agent 1'-(4-hexylphenyl)-[2,3'-bipyridin]-6'(1'*H*)-one (dot), respectively, within coordination polymer crystals of Cd−SNDI. Owing to the high boiling points of those two kinds of trapping reagents, the uptake and release amounts were monitored by time-course ^{1}H-NMR.

Supplementary Table 4. Variation of optimal conditions for photocleaving 1a by using different equiv. of electron donor.[a]

Entry	equiv. of electron donor	Yield (%)[b]
1	1.0 Equiv. of Electron Donor	11
2	8 Equiv. of Electron Donor	41
3	16 Equiv. of Electron Donor	58
4	24 Equiv. of Electron Donor	73
5	48 Equiv. of Electron Donor	85
6	8 Equiv. of Electron Donor, 24 hrs	80

[a]Reaction conditions: Ar–Cl **1a** (0.05 mmol, 1 equiv.), Cd–SNDI (10 mol%), DMF (3 mL), dibutylamine as electron donor, N_2 atmosphere, 455 nm LED, 40 °C, 4 hrs. [b]GC yields.

Supplementary Table 5. Detailed information on C–C and C–X bond formation reactions.[a]

Entry	Aryl Halide	Trapping Reagent	Targeted Product	No. of Compound	Isolated Yield (%)
1	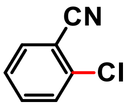	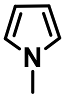	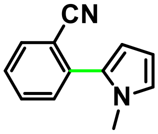	14	89
2	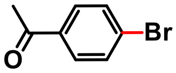	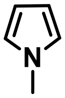	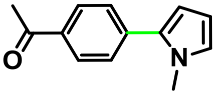	15	83
3	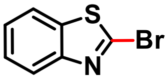	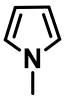	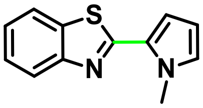	16	81
4	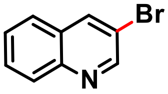	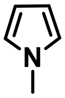	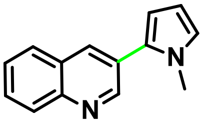	17	84
5	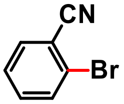	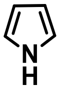	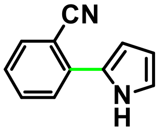	18	85
6	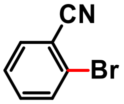	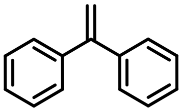	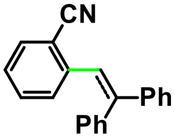 sat./unsat. = 2:1	19	62
7	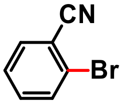	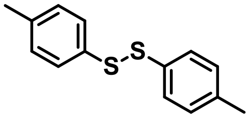	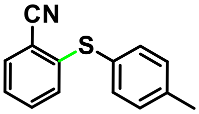	20	71
8	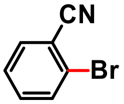	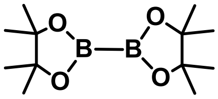	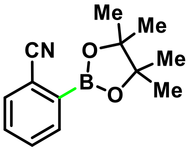	21	78
9	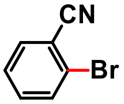	$P(OEt)_3$	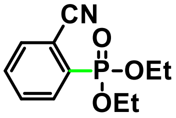	22	87
10	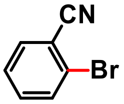	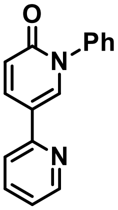	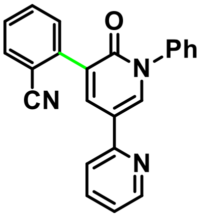	23	42

[a]All the aryl halides and trapping reagents were commercially available and used directly as received.

Characterisation of Tandem Photocleavage and C–C and C–X Bond Formation Reactions

The ^{1}H-NMR of C-C bond formation products **14** and **15** were in accordance with the reported data of our previous paper[26]. Thus, the detailed characterisation for these two compounds was omitted.

2-(1-Methyl-1*H*-pyrrol-2-yl)benzo[*d*]thiazole (**16**):

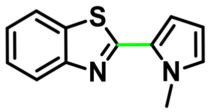

^{1}H-NMR (400 MHz, $CDCl_3$): δ 7.95 (d, J = 8.0 Hz, 1H), 7.83 (d, J = 8.0 Hz, 1H), 7.44 (t, J = 8.0 Hz, 1H), 7.32 (t, J = 8.0 Hz, 1H), 6.83 (t, J = 4.0 Hz, 2H), 6.21–6.22 (m, 1H), 4.16 (s, 3H).

^{13}C-NMR (126 MHz, $CDCl_3$): δ 160.5, 154.4, 134.0, 128.0, 126.6, 125.9, 124.5, 122.5, 121.1, 114.8, 108.8, 37.2.

This compound has been reported by literature, and our spectra were consistent with the literature[27].

3-(1-Methyl-1*H*-pyrrol-2-yl) quinoline (**17**):

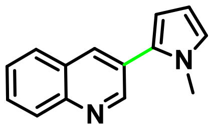

^{1}H-NMR (400 MHz, DMSO-d_6): δ 9.03 (d, J = 4.0 Hz, 1H), 8.12 (d, J = 8.0 Hz, 2H), 7.83 (d, J = 8.0 Hz, 1H), 7.71 (t, J = 8.0 Hz, 1H), 7.57 (t, J = 8.0 Hz, 1H), 6.82–6.83 (m, 1H), 6.42–6.43 (m, 1H), 6.29 (t, J = 4.0 Hz, 1H), 3.76 (s, 3H).

^{13}C-NMR (126 MHz, $CDCl_3$): δ 151.0, 146.7, 133.8, 131.0, 129.3, 129.2, 127.8, 127.7, 127.0, 126.5, 125.0, 110.3, 108.5, 35.2. This compound has been reported by literature, and our spectra were consistent with literature[27].

2-(1*H*-pyrrol-2-yl)benzonitrile (**18**):

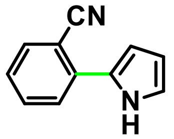

^{1}H-NMR (400 MHz, $CDCl_3$) δ 9.19 (br s, 1H), 7.63 (ddd, J = 8.1, 4.2, 1.3 Hz, 1H), 7.55 (ddd, J = 8.3, 7.3, 1.4 Hz, 1H), 7.24 (td, J = 7.6, 1.3 Hz, 1H), 6.97 (td, J = 2.8, 1.4 Hz, 1H), 6.82 (ddd, J = 4.0, 2.8, 1.5 Hz, 1H), 6.35 (dt, J = 3.7, 2.6 Hz, 1H).

^{13}C-NMR (126 MHz, $CDCl_3$) δ 135.9, 134.2, 133.3, 128.4, 126.8, 126.0, 121.0, 120.3, 110.55, 110.50, 106.2.

This compound has been reported by literature, and our spectra were consistent with the literature[27].

2-(2,2-Diphenylethyl)benzonitrile (sat.) and 2-(2,2-diphenylvinyl)benzonitrile (unsat.) (**19**):

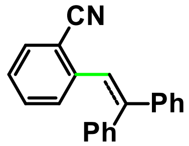

sat./unsat. = 2:1

These compounds were isolated as an inseparable mixture of saturated (sat.) and unsaturated (unsat.) compounds.

^{1}H-NMR (400 MHz, $CDCl_3$) δ 7.82 – 7.79 (m, 1H; unsat.), 7.71–7.68 (m, 1H; unsat.), 7.67 (dd, J = 7.5, 2.0 Hz, 1H; sat.), 7.62–7.57 (m, 1H; unsat.), 7.55 (dd, J = 7.7, 1.5 Hz, 1H; sat.), 7.51–7.39 (m, 2H; sat.), 7.36–7.32 (m, 2H; sat.), 7.32–7.27 (m, 4H; unsat.), 7.24 (d, J = 2.5 Hz, 4H; sat.), 7.23–7.20 (m, 5H; unsat.), 7.20–7.13 (m, 4H; sat.), 7.04 (dd, J = 7.9, 1.2 Hz, 2H; unsat.), 6.92–6.89 (m, 1H; unsat.), 4.37 (t, J = 8.0 Hz, 1H; sat.), 3.58 (d, J = 8.0 Hz, 2H; sat.).

^{13}C-NMR (101 MHz, $CDCl_3$) δ 144.2 (sat.), 143.3 (sat.), 142.4 (unsat.), 141.5 (unsat.), 139.5 (unsat.), 134.5 (unsat.), 134.0 (unsat.), 133.4 (sat.), 132.9 (sat.), 132.4 (sat.), 131.9 (unsat.), 130.6 (unsat.), 130.4 (sat.), 130.2 (unsat.), 130.0 (unsat.), 128.8 (unsat.), 128.6 (sat.), 128.5 (unsat.), 128.4 (unsat.), 128.2 (unsat.), 128.2 (sat.), 128.1 (unsat.), 127.8 (unsat.), 127.0 (unsat.), 126.8 (unsat.), 126.7 (sat.), 125.5 (unsat.), 123.7 (unsat.), 118.4 (unsat.), 113.0 (unsat.), 52.3 (sat.), 40.7 (sat.).

HRMS (EI) m/z calcd. for $C_{21}H_{17}N^+$ $[M_{sat.}]^+$ 283.1356, found 283.1350. HRMS (EI) m/z calcd. For $C_{21}H_{15}N^+$ $[M_{unsat.}]^+$ 281.1199, found 281.1185. This compound has been reported by literature, and our spectra were consistent with the literature[28].

2-(*p*-Tolylthio)benzonitrile (**20**)

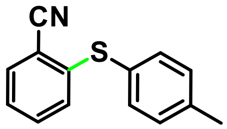

^{1}H-NMR (400 MHz, $CDCl_3$) δ 7.61 (dd, J = 7.7, 1.6 Hz, 1H), 7.42–7.32 (m, 3H), 7.24–7.18 (m, 3H), 7.03 (dd, J = 8.1, 1.1 Hz, 1H), 2.38 (s, 3H).

^{13}C-NMR (101 MHz, $CDCl_3$) δ 143.6, 139.7, 134.4, 133.6, 133.0, 130.8, 129.1, 127.8, 126.0, 117.1, 112.1, 21.4.

Our spectra of this compound were consistent with the literature[29].

1-(4-(4,4,5,5-Tetramethyl-1,3,2-dioxaborolan-2-yl)phenyl)ethanone (**21**):

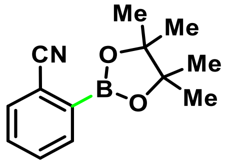

The compound was prepared according to the general procedure except for several changes: $CH_3CN:H_2O$ = 1.9 ml:0.1 ml as the solvent, the dose of electron donor dibutylamine and radical trapping agent $B_2(pin)_2$ are all 5 equiv.

^{1}H-NMR (400 MHz, $CDCl_3$): δ 7.88–7.94 (m, 4H), 2.62 (s, 3H), 1.36 (s, 12H).

^{13}C-NMR (126 MHz, $CDCl_3$): δ 198.5, 139.0, 134.9, 127.3, 84.2, 26.6, 24.9. The carbon directly attached to the boron atom was not detected due to quadrupolar broadening. Our spectra of this compound were consistent with the literature[30].

Diethyl (2-cyanophenyl)phosphonate (**22**):

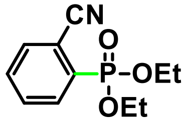

The compound was prepared according to the general procedure, except the dose of radical trapping agent $P(OEt)_3$ was 0.3 mmol.

^{1}H-NMR (400 MHz, $CDCl_3$): δ 8.13–8.08 (m, 1H), 7.81–7.78 (m, 1H), 7.69–7.62 (m, 2H), 4.30–4.16 (m, 4H), 1.37 (t, J = 8.0 Hz, 6H).

^{13}C-NMR (101 MHz, $CDCl_3$): δ 134.5, 133.2, 132.4, 132.2, 131.3, 117.1, 114.6, 63.2, 16.3. Our spectra of this compound were consistent with literature[27].

2-(6'-Oxo-1'-phenyl-1',6'-dihydro-[2,3'-bipyridin]-5'-yl)benzonitrile (**23**, known as Perampanel®):

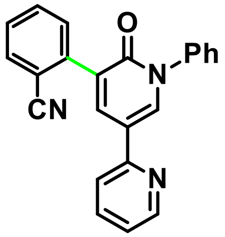

The compound was prepared according to the general procedure, except the amount of DMSO solvent was 3 mL.

^{1}H-NMR (400 MHz, $CDCl_3$) δ 8.59 (ddd, J = 4.8, 1.9, 0.9 Hz, 1H), 8.34–8.27 (m, 2H), 7.82–7.70 (m, 3H), 7.67–7.58 (m, 2H), 7.53 (virtual d, J = 4.3 Hz, 4H), 7.49–7.43 (m, 2H), 7.21 (ddd, J = 7.5, 4.8, 1.1 Hz, 1H).

^{13}C-NMR (126 MHz, $CDCl_3$) δ 160.6, 153.2, 149.8, 141.1, 140.5, 139.0, 138.2, 137.3, 133.3, 132.4, 131.2, 129.5, 129.1, 129.0, 128.4, 126.8, 122.2, 119.0, 118.7, 118.4, 112.6. The spectra were consistent with literature[31].

1'-(4-hexylphenyl)-[2,3'-bipyridin]-6'(1'*H*)-one'-(4-hexylphenyl)-[2,3'-bipyridin]-6'(1'*H*)-one (**Trapping Reagent S-2**)

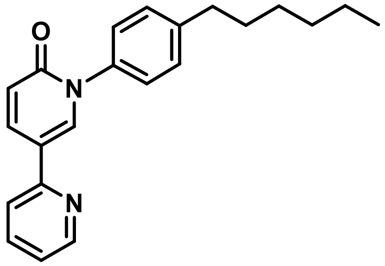

^{1}H NMR (600 MHz, $CDCl_3$) δ 8.57 (dd, *J* = 4.7, 1.6 Hz, 1H), 8.20 (d, *J* = 2.5 Hz, 1H), 8.04 (dd, *J* = 9.6, 2.6 Hz, 1H), 7.71 (td, *J* = 7.8, 1.8 Hz, 1H), 7.52 (d, *J* = 8.0 Hz, 1H), 7.34 (d, *J* = 8.1 Hz, 2H), 7.30 (d, *J* = 8.0 Hz, 2H), 7.18 (dd, *J* = 7.5, 4.8 Hz, 1H), 6.76 (d, *J* = 9.5 Hz, 1H), 2.66 (t, *J* = 7.8 Hz, 2H), 1.64 (p, *J* = 7.7, 7.3 Hz, 2H), 1.39 – 1.35 (m, 2H), 1.33 – 1.31 (m, 3H), 0.92 – 0.87 (m, 4H).

^{13}C NMR (101 MHz, $CDCl_3$) δ 162.4, 153.4, 149.8, 143.8, 138.6, 138.3, 137.7, 137.1, 129.4, 126.4, 122.0, 121.4, 118.58, 118.55, 35.7, 31.8, 31.4, 29.1, 22.7, 14.2.

Our spectra of this compound are consistent with literature[32].

¹H and ¹³C NMR spectra of the isolated compounds

5,5'-(4,9-Bis(ethylthio)-1,3,6,8-tetraoxo-1,3,6,8-tetrahydrobenzo[*lmn*][3,8]phenanthroline-2,7-diyl)diisophthalic acid (H_4SNDI) (recrystallized in HOAc under dark)

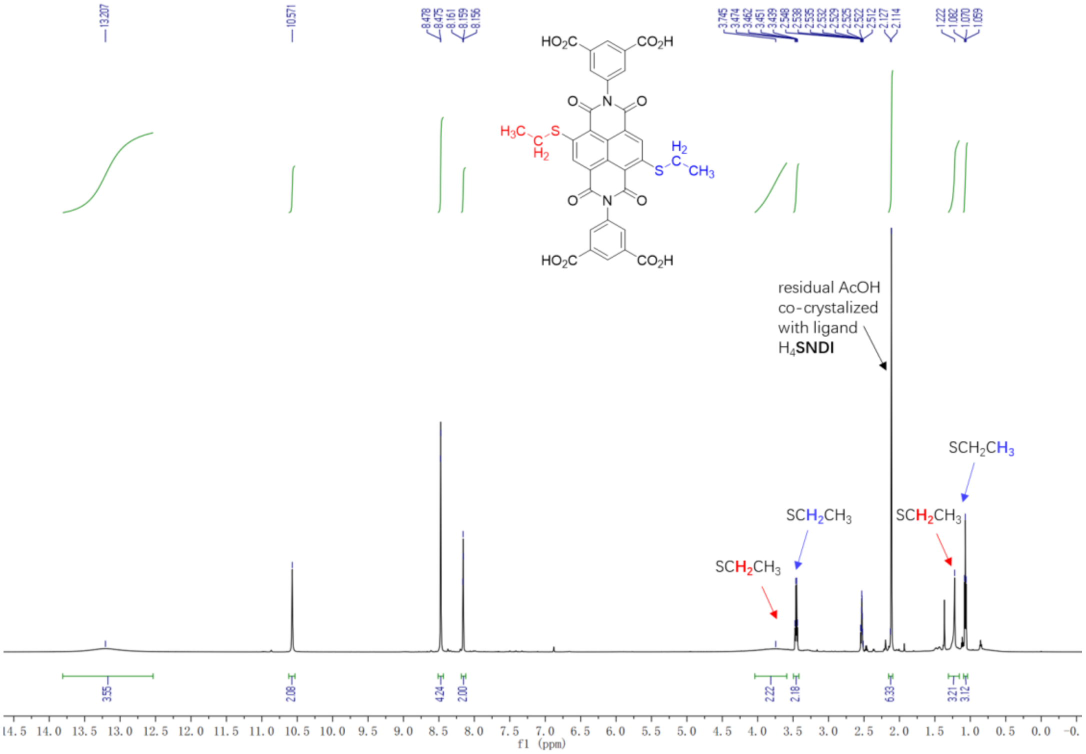

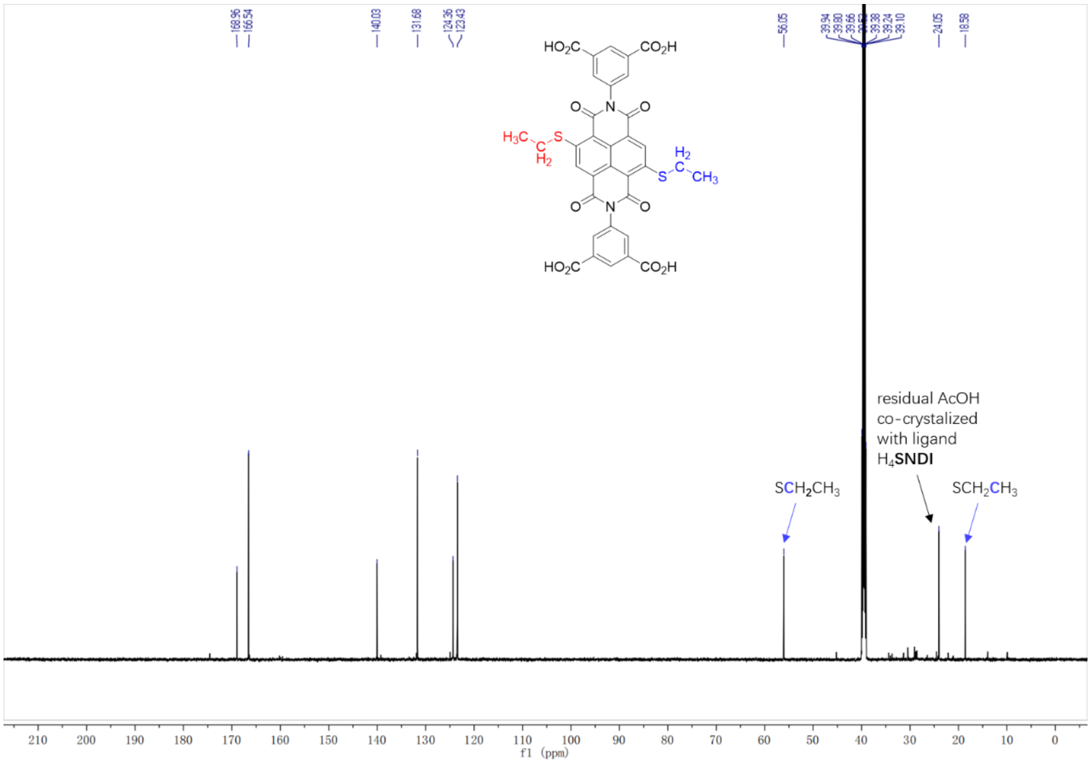

5,5'-(4,9-Bis(ethylthio)-1,3,6,8-tetraoxo-1,3,6,8-tetrahydrobenzo[*lmn*][3,8]phenanthroline-2,7-diyl)diisophthalic acid (H_4SNDI) (recrystallized in HOAc under the ambient daylight)

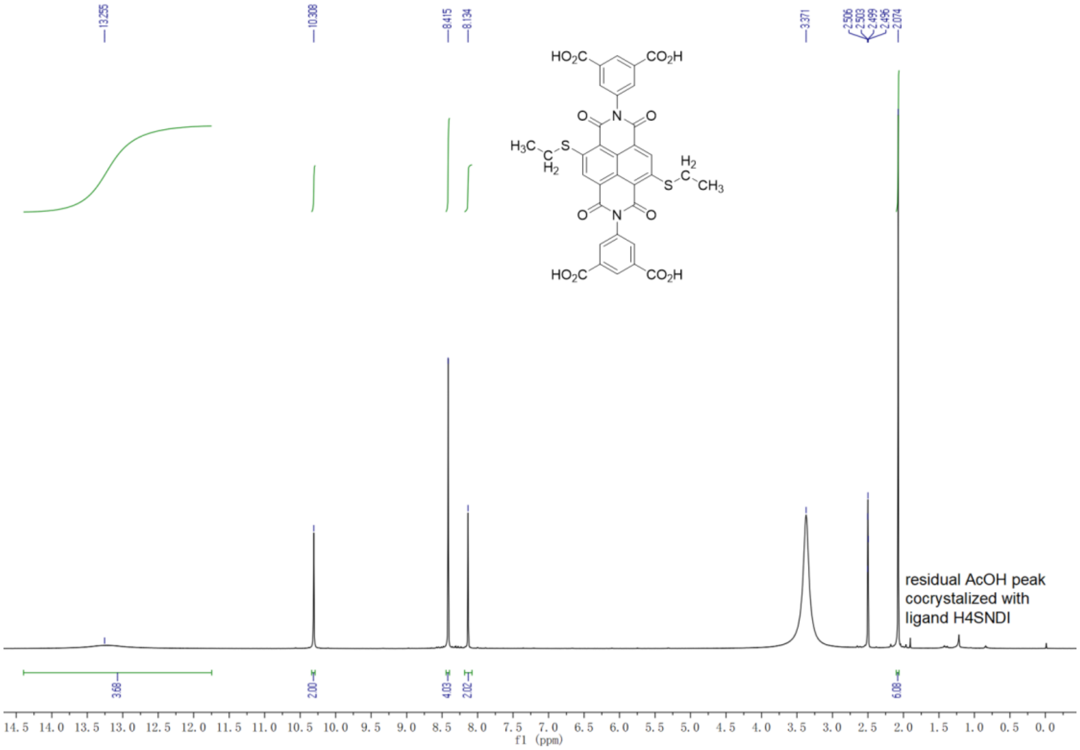

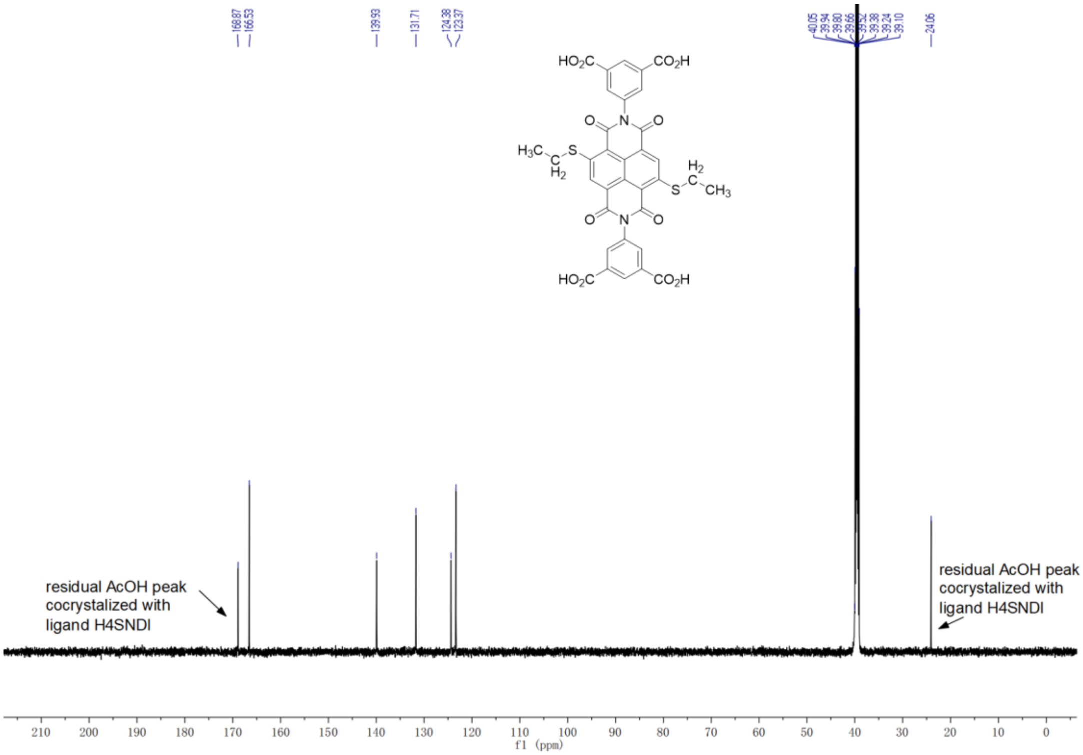

Tetramethyl 5,5'-(4,9-bis(ethylthio)-1,3,6,8-tetraoxo-1,3,6,8-tetrahydrobenzo[*lmn*][3,8]phenanthroline-2,7-diyl)diisophthalate (obtained from the esterification of ligand H_4SNDI with MeOH and the successive HOAc recrystallisation under the ambient daylight)

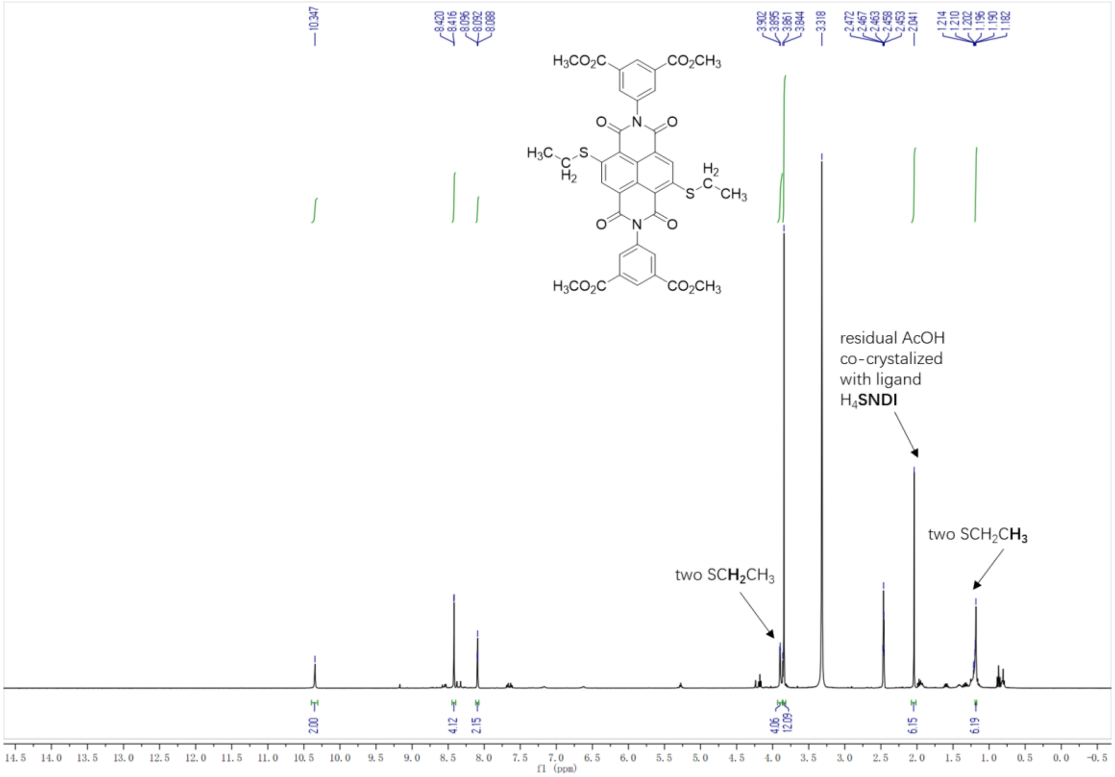

2-(1-Methyl-1*H*-pyrrol-2-yl)benzo[*d*]thiazole (**16**)

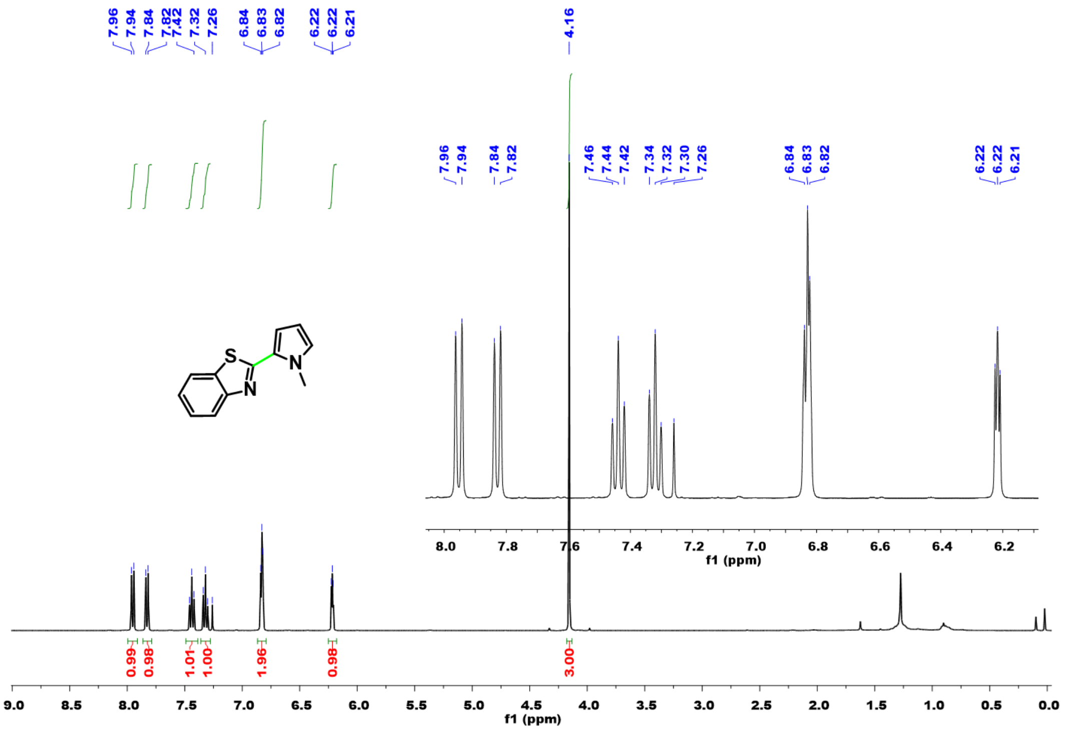

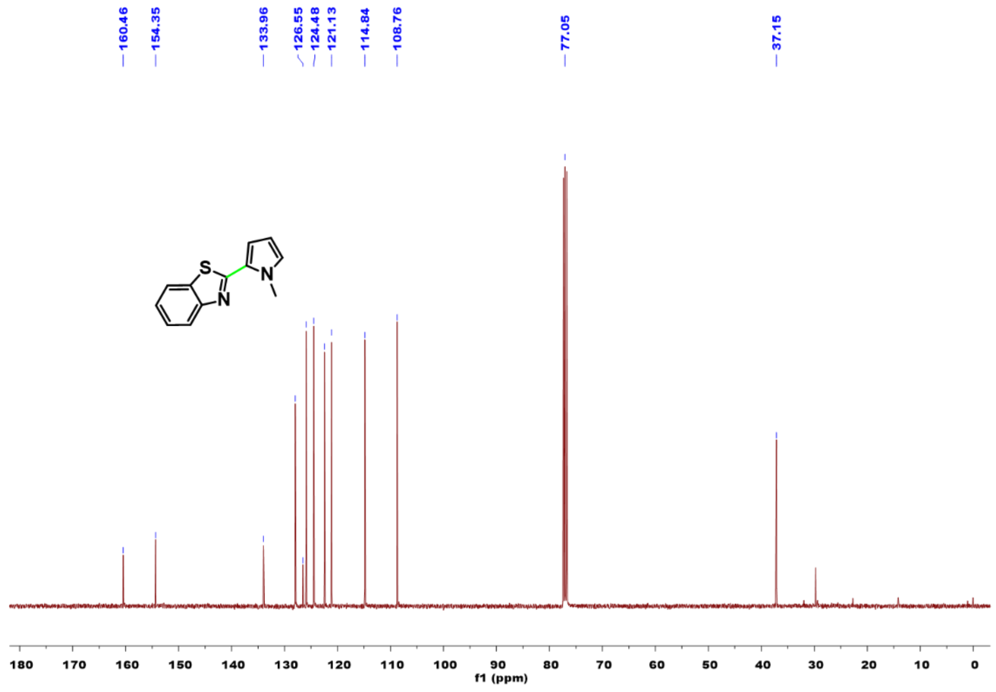

3-(1-Methyl-1*H*-pyrrol-2-yl)quinoline (**17**)

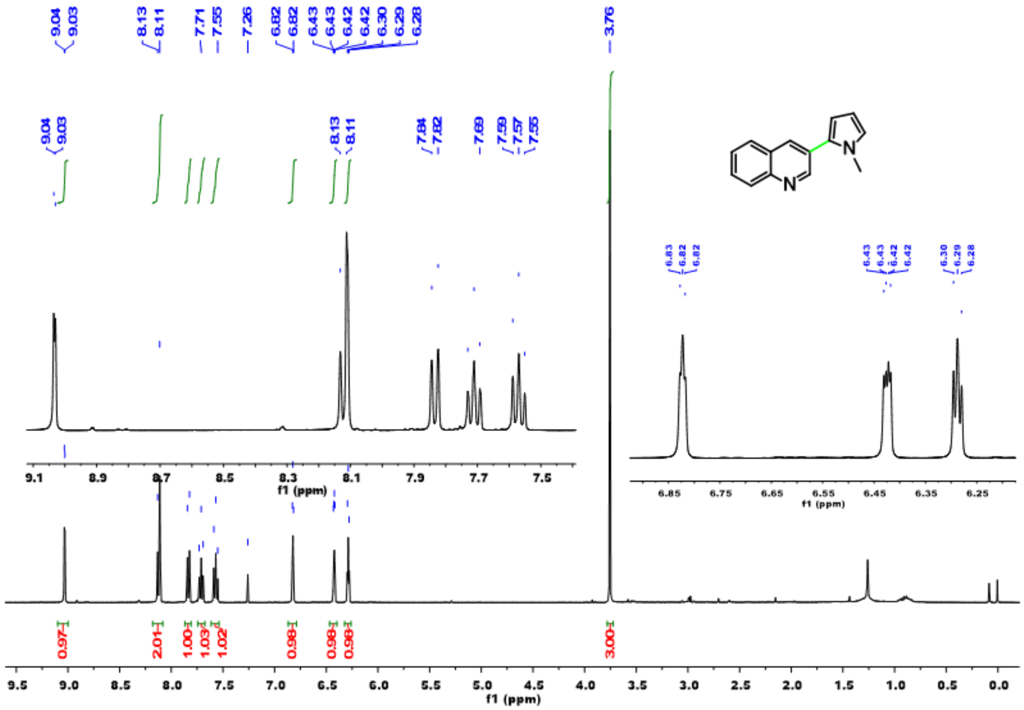

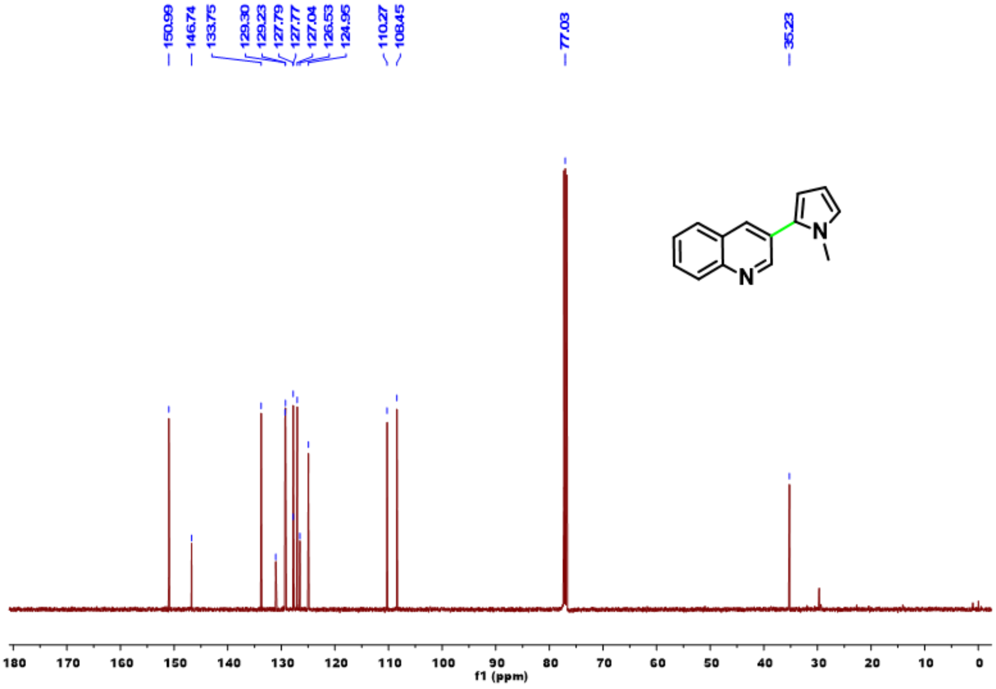

2-(1*H*-pyrrol-2-yl)benzonitrile (**18**)

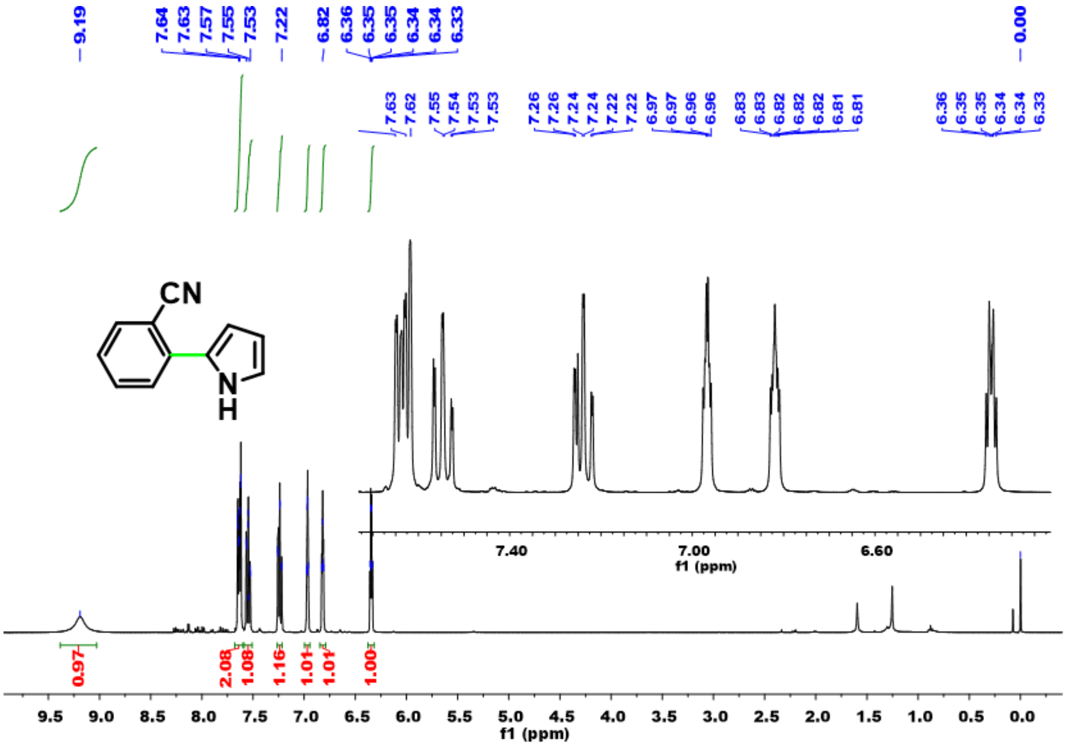

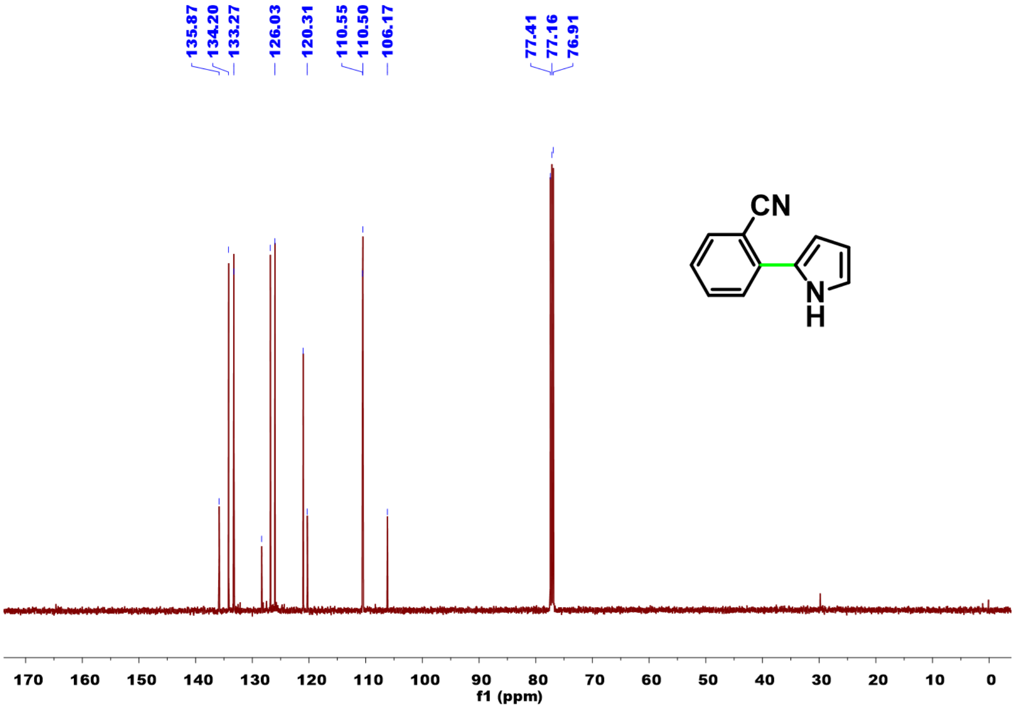

2-(2,2-Diphenylethyl)benzonitrile (sat.) and 2-(2,2-diphenylvinyl)benzonitrile (unsat.) (**19**)

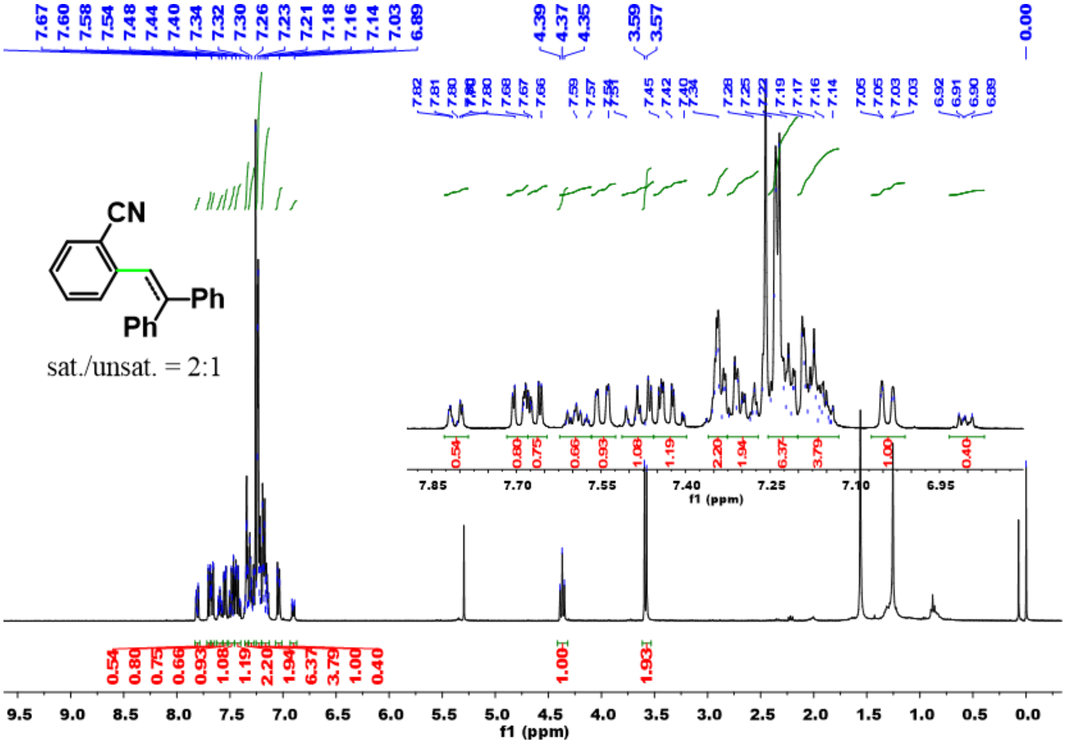

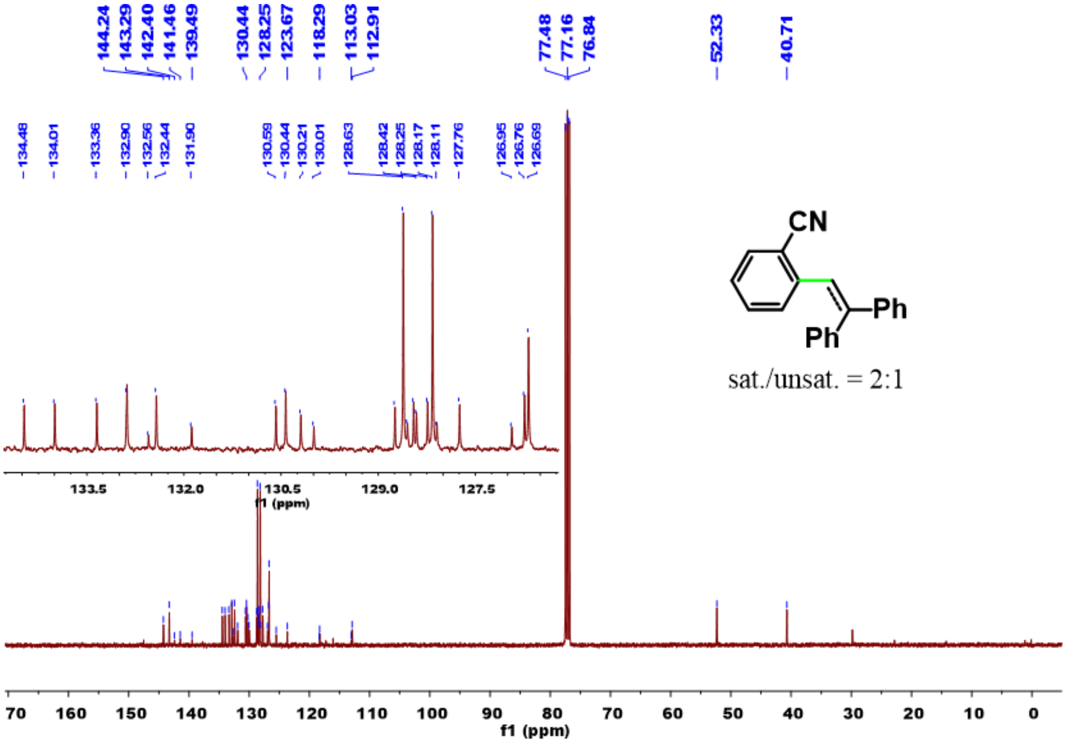

2-(*p*-Tolylthio)benzonitrile (**20**)

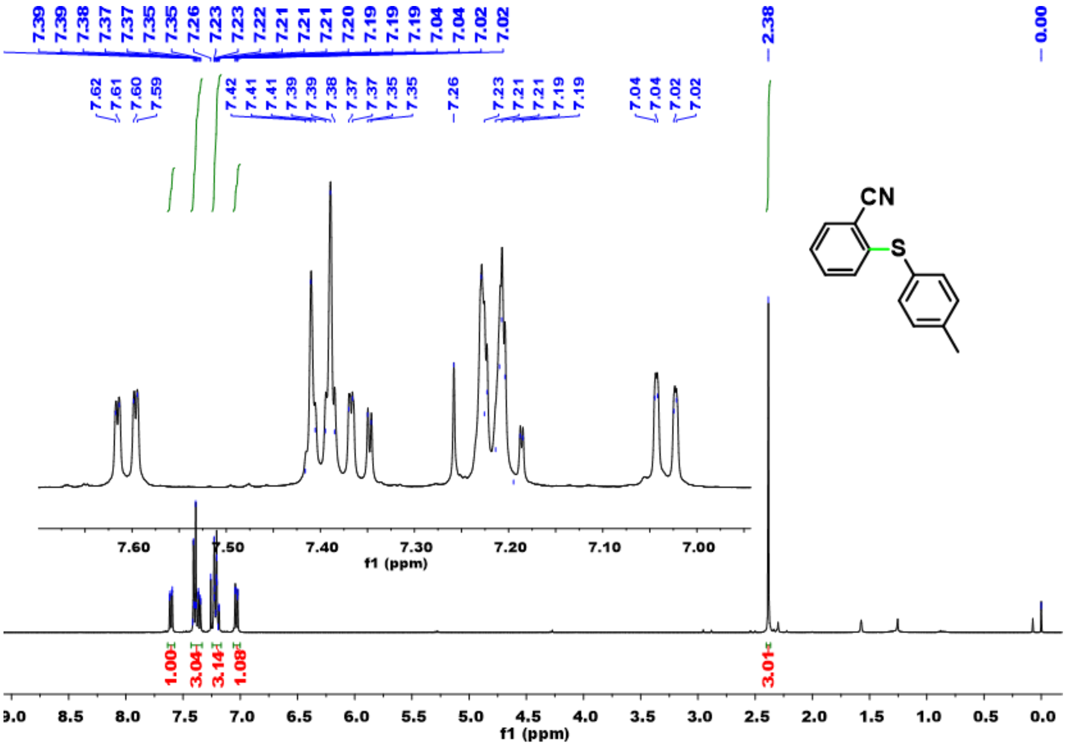

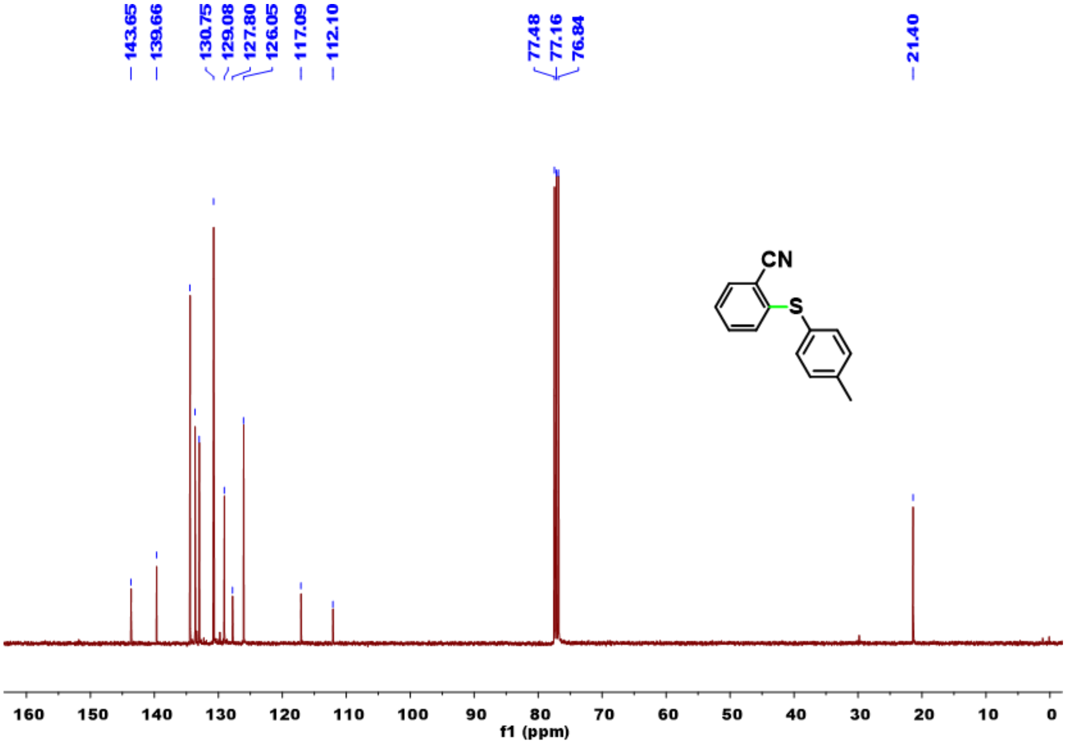

Diethyl (2-cyanophenyl)phosphonate (**21**)

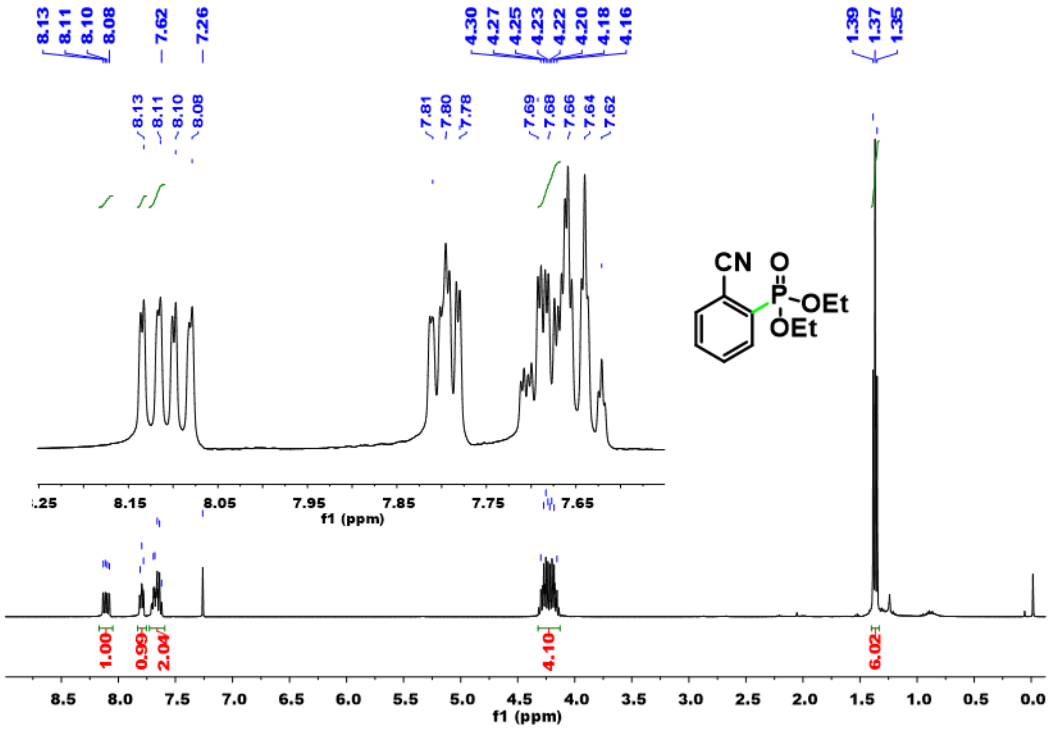

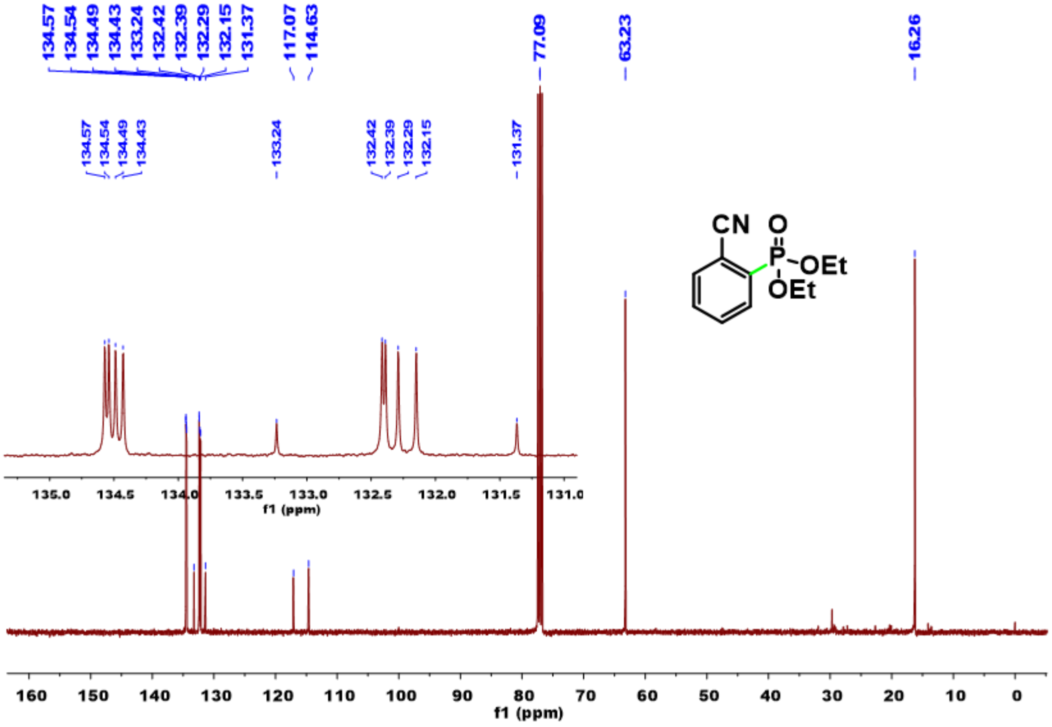

1-(4-(4,4,5,5-Tetramethyl-1,3,2-dioxaborolan-2-yl)phenyl)ethenone (**22**)

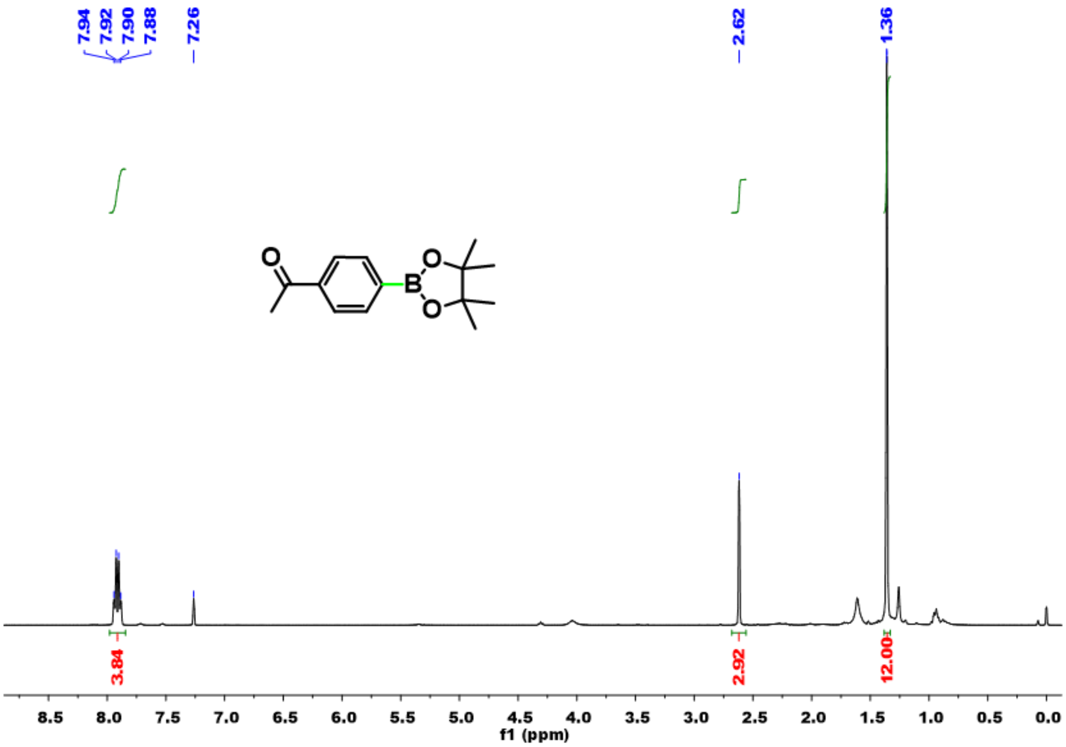

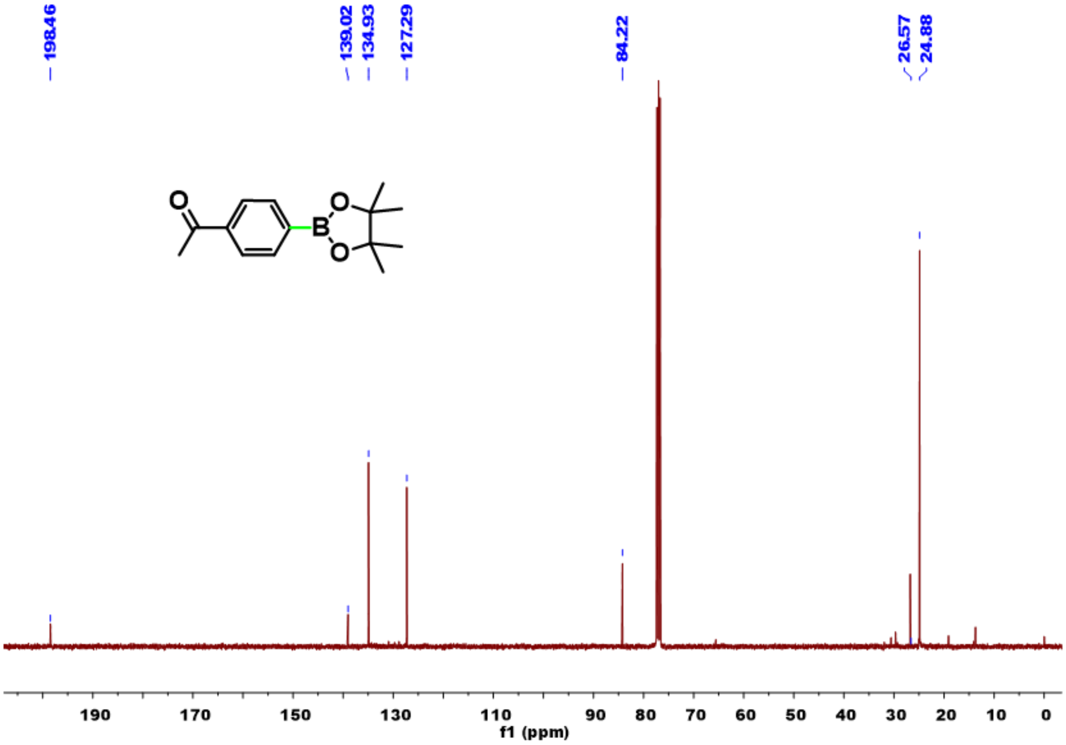

2-(6'-Oxo-1'-phenyl-1',6'-dihydro-[2,3'-bipyridin]-5'-yl)benzonitrile (**23**)

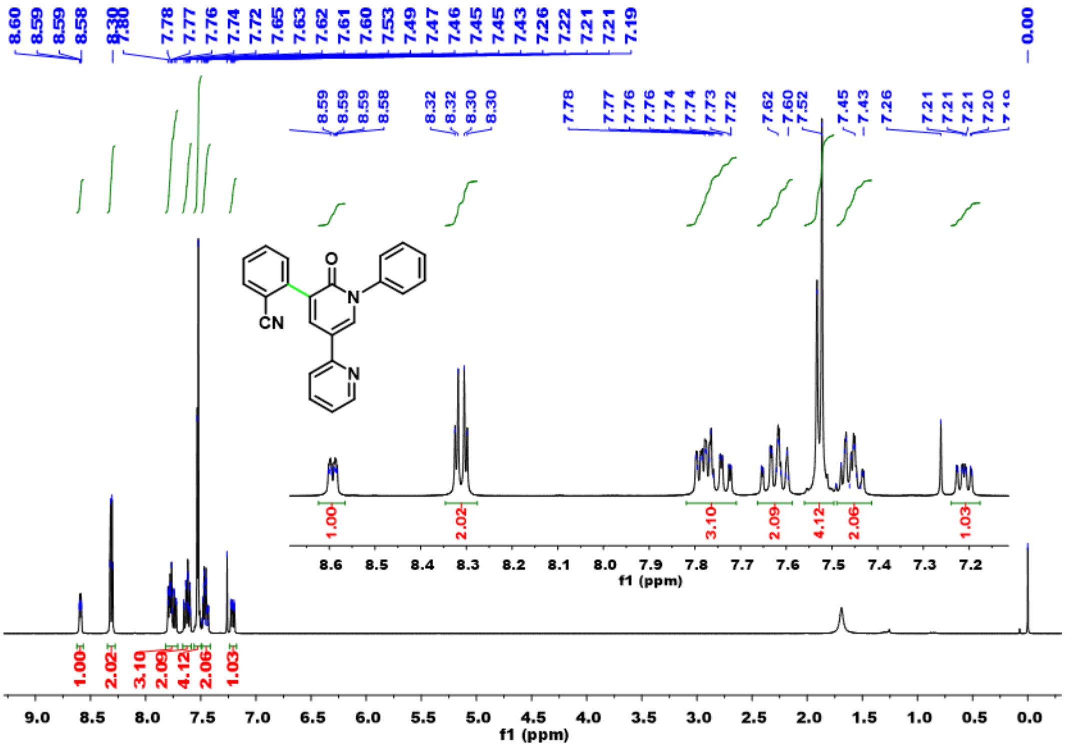

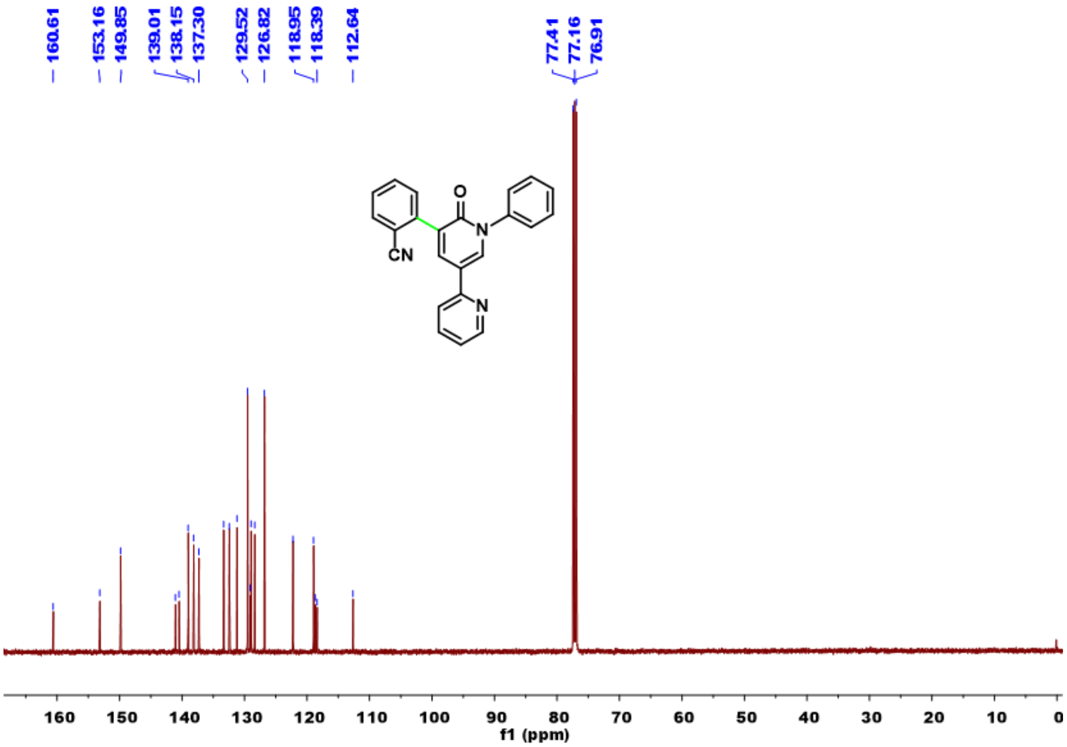

References

1 Ghosh, I., Ghosh, T., Bardagi, J. I. & Konig, B. Reduction of aryl halides by consecutive visible light-induced electron transfer processes. *Science* **346**, 725-728 (2014).

2 Zeman, C. J. t., Kim, S., Zhang, F. & Schanze, K. S. Direct Observation of the Reduction of Aryl Halides by a Photoexcited Perylene Diimide Radical Anion. *J. Am. Chem. Soc.* **142**, 2204-2207 (2020).

3 Discekici, E. H. *et al.* A highly reducing metal-free photoredox catalyst: design and application in radical dehalogenations. *Chem. Comm.* **51**, 11705-11708 (2015).

4 Cowper, N. G. W., Chernowsky, C. P., Williams, O. P. & Wickens, Z. K. Potent Reductants via Electron-Primed Photoredox Catalysis: Unlocking Aryl Chlorides for Radical Coupling. *J. Am. Chem. Soc.* **142**, 2093-2099 (2020).

5 Chmiel, A. F., Williams, O. P., Chernowsky, C. P., Yeung, C. S. & Wickens, Z. K. Non-innocent Radical Ion Intermediates in Photoredox Catalysis: Parallel Reduction Modes Enable Coupling of Diverse Aryl Chlorides. *J. Am. Chem. Soc.* **143**, 10882-10889 (2021).

6 Xu, J. *et al.* Unveiling Extreme Photoreduction Potentials of Donor–Acceptor Cyanoarenes to Access Aryl Radicals from Aryl Chlorides. *J. Am. Chem. Soc.* **143**, 13266-13273 (2021).

7 Kishore, R. S. K., Ravikumar, V., Bernardinelli, G., Sakai, N. & Matile, S. Rapid and Mild Synthesis of Functionalized Naphthalenediimides. *J. Org. Chem.* **73**, 738-740 (2008).

8 Guha, S. *et al.* Electronically Regulated Thermally and Light-Gated Electron Transfer from Anions to Naphthalenediimides. *J. Am. Chem. Soc.* **133**, 15256-15259 (2011).

9 Chowdhury, B. *et al.* Discriminative Behavior of a Donor–Acceptor–Donor Triad toward Cyanide and Fluoride: Insights into the Mechanism of Naphthalene Diimide Reduction by Cyanide and Fluoride. *Inorg. Chem.* **59**, 13371-13382 (2020).

10 Schmidt, S. B., Biskup, T., Jiao, X., McNeill, C. R. & Sommer, M. Controlling intermolecular redox-doping of naphthalene diimides. *J. Mater. Chem. C* **7**, 4466-4474 (2019).

11 Luo, X. *et al.* Triplet Energy Transfer from Perovskite Nanocrystals Mediated by Electron Transfer. *J. Am. Chem. Soc.* **142**, 11270-11278 (2020).

12 La Porte, N. T., Christensen, J. A., Krzyaniak, M. D., Rugg, B. K. & Wasielewski, M. R. Spin-Selective Photoinduced Electron Transfer within Naphthalenediimide Diradicals. *J. Phys. Chem. B* **123**, 7731-7739 (2019).

13 Lu, C., Fujitsuka, M., Sugimoto, A. & Majima, T. Dual Character of Excited Radical Anions in Aromatic Diimide Bis(radical anion)s: Donor or Acceptor? *J. Phys. Chem. C* **121**, 4558-4563 (2017).

14 Monney, N. P. A., Bally, T., Bhagavathy, G. S. & Glass, R. S. Spectroscopic Evidence for a New Type of Bonding between a Thioether Radical Cation and a Phenyl Group. *Org. Lett.* **15**, 4932-4935 (2013).

15 Senthil Murugan, K. *et al.* Visible-Light Activation of the Bimetallic Chromophore–Catalyst Dyad: Analysis of Transient Intermediates and Reactivity toward Organic Sulfides. *J. Phys. Chem. A.* **118**, 4451-4463 (2014).

16 Rajkumar, E. & Rajagopal, S. Photoinduced electron transfer reaction of tris(4,4'-dicarboxyl-2,2'-bipyridine)ruthenium(ii) ion with organic sulfides. *Photoch. Photobio. Sci.* **7**, 1407-1414 (2008).

17 Baciocchi, E., Del Giacco, T., Elisei, F. & Lapi, A. Sulfur Radical Cations. Kinetic and Product Study of the Photoinduced Fragmentation Reactions of (Phenylsulfanylalkyl)trimethylsilanes and Phenylsulfanylacetic Acid Radical Cations. *J. Org. Chem.* **71**, 853-860 (2006).

18 Lu, C., Fujitsuka, M., Sugimoto, A. & Majima, T. Excited-State Properties of Radical Anions of C70 and Its Derivatives: Significant Differences from the Case of C60. *J. Phys. Chem. C* **122**, 13385-13390 (2018).

19 Zhao, Z. *et al.* Visible Light Generation of a Microsecond Long-Lived Potent Reducing Agent. *J. Am. Chem. Soc.* **144**, 7043-7047 (2022).

20 Gosztola, D., Niemczyk, M. P., Svec, W., Lukas, A. S. & Wasielewski, M. R. Excited Doublet States of Electrochemically Generated Aromatic Imide and Diimide Radical Anions. *J. Phys. Chem. A.* **104**, 6545-6551 (2000).

21 Zhong, C. J., Kwan, W. S. V. & Miller, L. L. Self-assembly of delocalized .pi.-stacks in solution. Assessment of structural effects. *Chem. Mater.* **4**, 1423-1428 (1992).

22 Saha, M. & Bandyopadhyay, S. Stimuli Responsive Stable Radical Anion for Conductance Switching. *J. Phys. Chem. C* **125**, 6427-6432 (2021).

23 Park, S. S. *et al.* Cation-Dependent Intrinsic Electrical Conductivity in Isostructural Tetrathiafulvalene-Based Microporous Metal–Organic Frameworks. *J. Am. Chem. Soc.* **137**, 1774-1777 (2015).

24 Fujitsuka, M., Kim, S. S., Lu, C., Tojo, S. & Majima, T. Intermolecular and Intramolecular Electron Transfer Processes from Excited Naphthalene Diimide Radical Anions. *J. Phys. Chem. B* **119**, 7275-7282 (2015).

25 Lu, C., Fujitsuka, M., Sugimoto, A. & Majima, T. Unprecedented Intramolecular Electron Transfer from Excited Perylenediimide Radical Anion. *J. Phys. Chem. C* **120**, 12734-12741 (2016).

26 Zeng, L. *et al.* Organized Aggregation Makes Insoluble Perylene Diimide Efficient for the Reduction of Aryl Halides via Consecutive Visible Light-Induced Electron-Transfer Processes. *J. Am. Chem. Soc.* **138**, 3958-3961 (2016).

27 Ghosh, I., Shaikh, R. S. & König, B. Sensitization-Initiated Electron Transfer for Photoredox Catalysis. *Angew. Chem. Int. Ed.* **56**, 8544-8549 (2017).

28 Ghosh, I. & König, B. Chromoselective Photocatalysis: Controlled Bond Activation through Light-Color Regulation of Redox Potentials. *Angew. Chem. Int. Ed.* **55**, 7676-7679 (2016).
29 Liu, B., Lim, C. H. & Miyake, G. M. Visible-Light-Promoted C-S Cross-Coupling via Intermolecular Charge Transfer. *J. Am. Chem. Soc.* **139**, 13616-13619 (2017).
30 Jiang, M., Yang, H. & Fu, H. Visible-Light Photoredox Borylation of Aryl Halides and Subsequent Aerobic Oxidative Hydroxylation. *Org. Lett.* **18**, 5248-5251 (2016).
31 Lee, H. G., Milner, P. J., Placzek, M. S., Buchwald, S. L. & Hooker, J. M. Virtually Instantaneous, Room-Temperature C-11 -Cyanation Using Biaryl Phosphine Pd(0) Complexes. *J. Am. Chem. Soc.* **137**, 648-651 (2015).
32 Hibi, S. et al. Discovery of 2-(2-Oxo-1-phenyl-5-pyridin-2-yl-1,2-dihydropyridin-3-yl)benzonitrile (Perampanel): A Novel, Noncompetitive α-Amino-3-hydroxy-5-methyl-4-isoxazolepropanoic Acid (AMPA) Receptor Antagonist. *J. Med. Chem.* **55**, 23, 10584–10600 (2012).
