# Supplementary material for: Chalcogen-bridged coordination polymer for the photocatalytic activation of aryl halides
Source: Nat Commun. 2023 Jul 6;14:4002. doi: 10.1038/s41467-023-39540-z (PMC10326065; doi:10.1038/s41467-023-39540-z)
Supplement: Supplementary file 2 — Description of Additional Supplementary Files [file 41467_2023_39540_MOESM2_ESM.pdf]

Description of Additional Supplementary Files

File Name: Supplementary Data 1
Description: checkCIF of Cd-SNDI

File Name: Supplementary Data 2
Description: checkCIF of Cd-NDI

File Name: Supplementary Data 3
Description: checkCIF of Pyrene@Sr-NDI
