# Supplementary material for: Chalcogen-bridged coordination polymer for the photocatalytic activation of aryl halides
Source: Nat Commun. 2023 Jul 6;14:4002. doi: 10.1038/s41467-023-39540-z (PMC10326065; doi:10.1038/s41467-023-39540-z)
Supplement: Supplementary file 3 — Supplementary Data 1-3 [file 41467_2023_39540_MOESM3_ESM.zip › Explanations of Any A- or B-Level Alerts.docx]

After refining cif file of Cd–SNDI to improve the quality of data analysis, the residual Level-B Alerts were listed and explained as below:

**Alert level B:**

(1) PLAT430_ALERT_2_B Short Inter D...A Contact O1 ..O301 . 2.63 Ang. y,1-x,-z = 3_565 Check

(2) PLAT430_ALERT_2_B Short Inter D...A Contact O1 ..N301 . 2.75 Ang. y,1-x,-z = 3_565 Check

(3) PLAT936_ALERT_2_B The Embedded .res File Includes a DAMP Command. 500.0 Report

**Explanation to Alert (1) & (2):** Atom O1 belongs to the carboxylate of Cd-SNDI, O301 and N301 belong to the lattice solvent DMF with partial occupation and structural disorder. These two alerts might be caused by spatial proximity of solvent DMF to the framework.

**Explanation to Alert (3):** The DAMP Command was used to stabilize the refinement.
