# Supplementary material for: Chalcogen-bridged coordination polymer for the photocatalytic activation of aryl halides
Source: Nat Commun. 2023 Jul 6;14:4002. doi: 10.1038/s41467-023-39540-z (PMC10326065; doi:10.1038/s41467-023-39540-z)

checkCIF/PLATON report

You have not supplied any structure factors. As a result the full set of tests cannot be run.

THIS REPORT IS FOR GUIDANCE ONLY. IF USED AS PART OF A REVIEW PROCEDURE FOR PUBLICATION, IT SHOULD NOT REPLACE THE EXPERTISE OF AN EXPERIENCED CRYSTALLOGRAPHIC REFEREE.

No syntax errors found. CIF dictionary Interpreting this report

Datablock: new3_sq_s

Bond precision: C-C = 0.0090 A Wavelength=0.71073

Cell:	a=10.0891(7)	b=10.0891(7)	c=35.288(2)
	alpha=90	beta=90	gamma=90
Temperature:	200 K		

	Calculated	Reported
Volume	3592.0(5)	3592.0(6)
Space group	P -4 2 c	P -4 2 c
Hall group	P -4 2c	P -4 2c
Moiety formula	4(C19.25 H13.25 Cd0.50 N1.75 O6.75 S), 3(C3 H7 N O), C H4 O [+	?
Sum formula	C87 H78 Cd2 N10 O31 S4 [+ solvent]	C43.50 H39 Cd N5 O15.50 S2
Mr	2112.65	1056.31
Dx,g cm-3	0.977	0.977
Z	1	2
Mu (mm-1)	0.410	0.410
F000	1078.0	1078.0
F000'	1077.42	
h,k,lmax	11,11,41	11,11,41
Nref	3154[1802]	3106
Tmin,Tmax		
Tmin'		

Correction method= Not given

Data completeness= 1.72/0.98 Theta(max)= 24.986

R(reflections)= 0.1051(1722) wR2(reflections)= 0.2963(3106)

S = 0.971 Npar= 195

The following ALERTS were generated. Each ALERT has the format
test-name_ALERT_alert-type_alert-level.
Click on the hyperlinks for more details of the test.

Alert level B

```
PLAT430_ALERT_2_B Short Inter D...A Contact  O1        ..O301     .       2.63 Ang.
                                                y,1-x,-z  =      3_565 Check
PLAT430_ALERT_2_B Short Inter D...A Contact  O1        ..N301     .       2.75 Ang.
                                                y,1-x,-z  =      3_565 Check
PLAT936_ALERT_2_B The Embedded .res File Includes a DAMP Command .      500.0 Report
```

Alert level C

```
STRVA01_ALERT_4_C           Flack test results are ambiguous.
           From the CIF: _refine_ls_abs_structure_Flack    0.490
           From the CIF: _refine_ls_abs_structure_Flack_su    0.170
PLAT029_ALERT_3_C _diffrn_measured_fraction_theta_full value Low .      0.977 Why?
PLAT052_ALERT_1_C Info on Absorption Correction Method   Not Given     Please Do !
PLAT053_ALERT_1_C Minimum Crystal Dimension Missing (or Error) ...     Please Check
PLAT054_ALERT_1_C Medium  Crystal Dimension Missing (or Error) ...     Please Check
PLAT055_ALERT_1_C Maximum Crystal Dimension Missing (or Error) ...     Please Check
PLAT082_ALERT_2_C High R1 Value ................................        0.11 Report
PLAT084_ALERT_3_C High wR2 Value (i.e. > 0.25) ..................        0.30 Report
PLAT220_ALERT_2_C NonSolvent   Resd 1   C    Ueq(max)/Ueq(min) Range      3.3 Ratio
PLAT220_ALERT_2_C NonSolvent   Resd 1   O    Ueq(max)/Ueq(min) Range      3.3 Ratio
PLAT241_ALERT_2_C High   'MainMol' Ueq as Compared to Neighbors of        N1 Check
PLAT241_ALERT_2_C High   'MainMol' Ueq as Compared to Neighbors of        C4 Check
PLAT242_ALERT_2_C Low    'MainMol' Ueq as Compared to Neighbors of        C5 Check
PLAT242_ALERT_2_C Low    'MainMol' Ueq as Compared to Neighbors of        C6 Check
PLAT250_ALERT_2_C Large U3/U1 Ratio for Average U(i,j) Tensor ....      2.1 Note
PLAT260_ALERT_2_C Large Average Ueq of Residue Including       Cd1      0.133 Check
PLAT309_ALERT_2_C Single Bonded Oxygen (C-O > 1.3 Ang) ..........       O301 Check
PLAT342_ALERT_3_C Low Bond Precision on  C-C Bonds ..............      0.009 Ang.
```

Alert level G

```
PLAT002_ALERT_2_G Number of Distance or Angle Restraints on AtSite         24 Note
PLAT003_ALERT_2_G Number of Uiso or Uij Restrained non-H Atoms ...         27 Report
PLAT004_ALERT_5_G Polymeric Structure Found with Maximum Dimension          3 Info
PLAT007_ALERT_5_G Number of Unrefined Donor-H Atoms .............          2 Report
PLAT045_ALERT_1_G Calculated and Reported Z Differ by a Factor ...      0.500 Check
PLAT172_ALERT_4_G The CIF-Embedded .res File Contains DFIX Records         14 Report
PLAT174_ALERT_4_G The CIF-Embedded .res File Contains FLAT Records          1 Report
PLAT178_ALERT_4_G The CIF-Embedded .res File Contains SIMU Records          3 Report
PLAT186_ALERT_4_G The CIF-Embedded .res File Contains ISOR Records          1 Report
PLAT188_ALERT_3_G A Non-default SIMU Restraint Value has been used     0.0200 Report
PLAT188_ALERT_3_G A Non-default SIMU Restraint Value has been used     0.0100 Report
PLAT188_ALERT_3_G A Non-default SIMU Restraint Value has been used     0.0200 Report
PLAT300_ALERT_4_G Atom Site Occupancy of S1          Constrained at       0.5 Check
PLAT300_ALERT_4_G Atom Site Occupancy of O401        Constrained at     0.375 Check
```

```
PLAT300_ALERT_4_G Atom Site Occupancy of C10        Constrained at        0.5 Check
PLAT300_ALERT_4_G Atom Site Occupancy of N401       Constrained at      0.375 Check
PLAT300_ALERT_4_G Atom Site Occupancy of C10'       Constrained at       0.25 Check
PLAT300_ALERT_4_G Atom Site Occupancy of C11        Constrained at       0.25 Check
PLAT300_ALERT_4_G Atom Site Occupancy of C11'       Constrained at       0.25 Check
PLAT300_ALERT_4_G Atom Site Occupancy of C401       Constrained at      0.375 Check
PLAT300_ALERT_4_G Atom Site Occupancy of C402       Constrained at      0.375 Check
PLAT300_ALERT_4_G Atom Site Occupancy of C403       Constrained at      0.375 Check
PLAT300_ALERT_4_G Atom Site Occupancy of H10A       Constrained at       0.25 Check
PLAT300_ALERT_4_G Atom Site Occupancy of H10B       Constrained at       0.25 Check
PLAT300_ALERT_4_G Atom Site Occupancy of H10C       Constrained at       0.25 Check
PLAT300_ALERT_4_G Atom Site Occupancy of H10D       Constrained at       0.25 Check
PLAT300_ALERT_4_G Atom Site Occupancy of H11A       Constrained at       0.25 Check
PLAT300_ALERT_4_G Atom Site Occupancy of H11B       Constrained at       0.25 Check
PLAT300_ALERT_4_G Atom Site Occupancy of H11C       Constrained at       0.25 Check
PLAT300_ALERT_4_G Atom Site Occupancy of H11D       Constrained at       0.25 Check
PLAT300_ALERT_4_G Atom Site Occupancy of H11E       Constrained at       0.25 Check
PLAT300_ALERT_4_G Atom Site Occupancy of H11F       Constrained at       0.25 Check
PLAT300_ALERT_4_G Atom Site Occupancy of H40A       Constrained at      0.375 Check
PLAT300_ALERT_4_G Atom Site Occupancy of H40B       Constrained at      0.375 Check
PLAT300_ALERT_4_G Atom Site Occupancy of H40C       Constrained at      0.375 Check
PLAT300_ALERT_4_G Atom Site Occupancy of H40D       Constrained at      0.375 Check
PLAT300_ALERT_4_G Atom Site Occupancy of H40E       Constrained at      0.375 Check
PLAT300_ALERT_4_G Atom Site Occupancy of H40F       Constrained at      0.375 Check
PLAT300_ALERT_4_G Atom Site Occupancy of H40G       Constrained at      0.375 Check
PLAT300_ALERT_4_G Atom Site Occupancy of O301       Constrained at      0.375 Check
PLAT300_ALERT_4_G Atom Site Occupancy of N301       Constrained at      0.375 Check
PLAT300_ALERT_4_G Atom Site Occupancy of C301       Constrained at      0.375 Check
PLAT300_ALERT_4_G Atom Site Occupancy of C302       Constrained at      0.375 Check
PLAT300_ALERT_4_G Atom Site Occupancy of C303       Constrained at      0.375 Check
PLAT300_ALERT_4_G Atom Site Occupancy of H30A       Constrained at      0.375 Check
PLAT300_ALERT_4_G Atom Site Occupancy of H30B       Constrained at      0.375 Check
PLAT300_ALERT_4_G Atom Site Occupancy of H30C       Constrained at      0.375 Check
PLAT300_ALERT_4_G Atom Site Occupancy of H30D       Constrained at      0.375 Check
PLAT300_ALERT_4_G Atom Site Occupancy of H30E       Constrained at      0.375 Check
PLAT300_ALERT_4_G Atom Site Occupancy of H30F       Constrained at      0.375 Check
PLAT300_ALERT_4_G Atom Site Occupancy of H30G       Constrained at      0.375 Check
PLAT300_ALERT_4_G Atom Site Occupancy of O100       Constrained at      0.125 Check
PLAT300_ALERT_4_G Atom Site Occupancy of C100       Constrained at      0.125 Check
PLAT300_ALERT_4_G Atom Site Occupancy of H10E       Constrained at      0.125 Check
PLAT300_ALERT_4_G Atom Site Occupancy of H10F       Constrained at      0.125 Check
PLAT300_ALERT_4_G Atom Site Occupancy of H10G       Constrained at      0.125 Check
PLAT300_ALERT_4_G Atom Site Occupancy of H10H       Constrained at      0.125 Check
PLAT301_ALERT_3_G Main Residue  Disorder .............(Resd  1  )        22% Note
PLAT302_ALERT_4_G Anion/Solvent/Minor-Residue Disorder (Resd  2  )       100% Note
PLAT302_ALERT_4_G Anion/Solvent/Minor-Residue Disorder (Resd  3  )       100% Note
PLAT367_ALERT_2_G Long?  C(sp?)-C(sp?) Bond  C1       - C3       .       1.52 Ang.
PLAT432_ALERT_2_G Short Inter X...Y Contact  O2       ..C100     .       2.89 Ang.
                                                -1+x,y,z  =      1_455 Check
PLAT432_ALERT_2_G Short Inter X...Y Contact  O2       ..C301     .       2.95 Ang.
                                                -1+x,y,z  =      1_455 Check
PLAT605_ALERT_4_G Largest Solvent Accessible VOID in the Structure        262 A**3
PLAT764_ALERT_4_G Overcomplete CIF Bond List Detected (Rep/Expd) .       1.17 Ratio
PLAT773_ALERT_2_G Check long C-C Bond in CIF: C9       --C11             1.88 Ang.
PLAT773_ALERT_2_G Check long C-C Bond in CIF: C11      --C11             1.80 Ang.
PLAT780_ALERT_1_G Coordinates do not Form a Properly Connected Set     Please Do !
PLAT811_ALERT_5_G No ADDSYM Analysis: Too Many Excluded Atoms ....          ! Info
```

```
PLAT860_ALERT_3_G Number of Least-Squares Restraints ............        408 Note
PLAT869_ALERT_4_G ALERTS Related to the Use of SQUEEZE  Suppressed         ! Info
PLAT883_ALERT_1_G No Info/Value for _atom_sites_solution_primary .     Please Do !
PLAT899_ALERT_4_G SHELXL2018 is Deprecated and Succeeded by SHELXL     2019/3 Note
PLAT933_ALERT_2_G Number of HKL-OMIT Records in Embedded .res File         38 Note
PLAT967_ALERT_5_G Note: Two-Theta Cutoff Value in Embedded .res ..       50.0 Degree
```

```
   0 ALERT level A = Most likely a serious problem - resolve or explain
   3 ALERT level B = A potentially serious problem, consider carefully
  18 ALERT level C = Check. Ensure it is not caused by an omission or oversight
  75 ALERT level G = General information/check it is not something unexpected

   7 ALERT type 1 CIF construction/syntax error, inconsistent or missing data
  21 ALERT type 2 Indicator that the structure model may be wrong or deficient
   8 ALERT type 3 Indicator that the structure quality may be low
  56 ALERT type 4 Improvement, methodology, query or suggestion
   4 ALERT type 5 Informative message, check
```

It is advisable to attempt to resolve as many as possible of the alerts in all categories. Often the minor alerts point to easily fixed oversights, errors and omissions in your CIF or refinement strategy, so attention to these fine details can be worthwhile. In order to resolve some of the more serious problems it may be necessary to carry out additional measurements or structure refinements. However, the purpose of your study may justify the reported deviations and the more serious of these should normally be commented upon in the discussion or experimental section of a paper or in the "special_details" fields of the CIF. checkCIF was carefully designed to identify outliers and unusual parameters, but every test has its limitations and alerts that are not important in a particular case may appear. Conversely, the absence of alerts does not guarantee there are no aspects of the results needing attention. It is up to the individual to critically assess their own results and, if necessary, seek expert advice.

Publication of your CIF in IUCr journals

A basic structural check has been run on your CIF. These basic checks will be run on all CIFs submitted for publication in IUCr journals (*Acta Crystallographica*, *Journal of Applied Crystallography*, *Journal of Synchrotron Radiation*); however, if you intend to submit to *Acta Crystallographica Section C* or *E* or *IUCrData*, you should make sure that full publication checks are run on the final version of your CIF prior to submission.

Publication of your CIF in other journals

Please refer to the *Notes for Authors* of the relevant journal for any special instructions relating to CIF submission.

PLATON version of 10/05/2023; check.def file version of 10/05/2023

Datablock new3_sq_s - ellipsoid plot

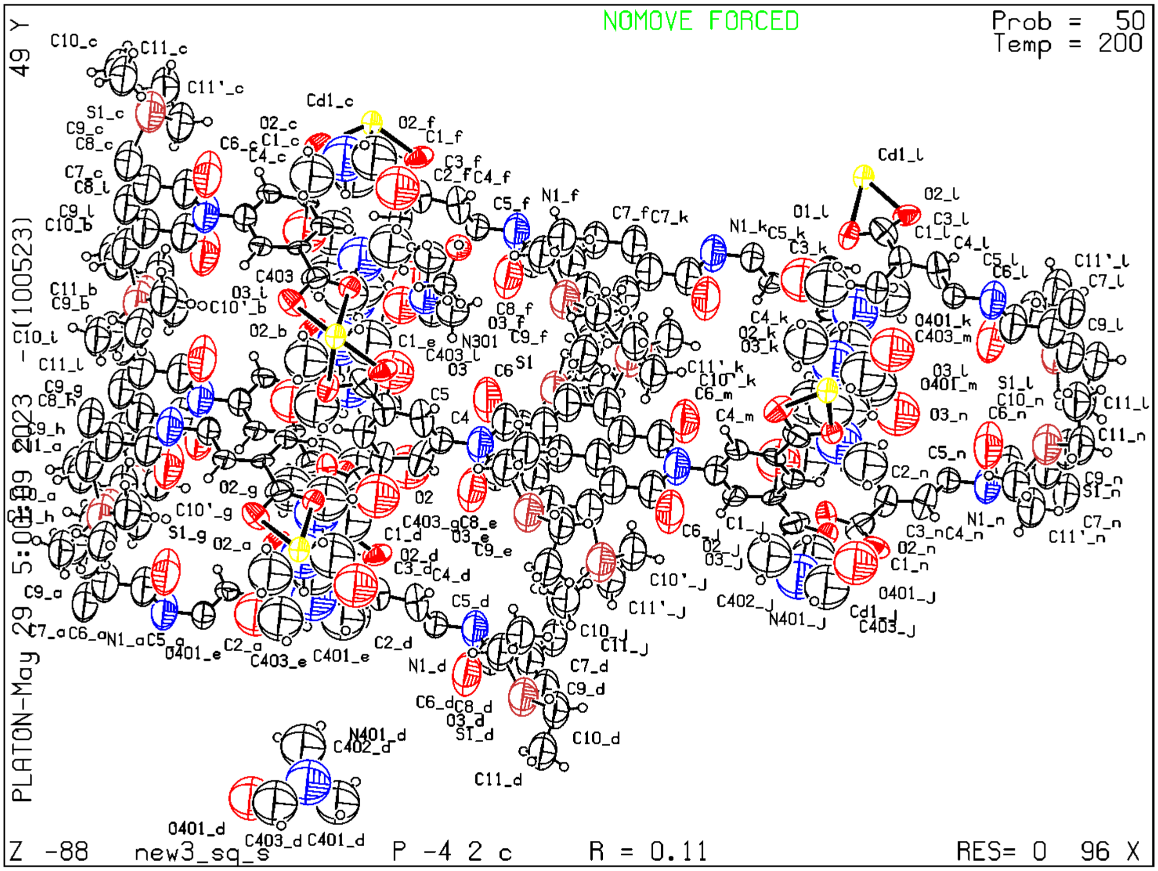

Supplement: Supplementary file 3 — Supplementary Data 1-3 [file 41467_2023_39540_MOESM3_ESM.zip › Supplementary Data 1-checkCIF of Cd-SNDI.pdf]
