# Supplementary material for: Chalcogen-bridged coordination polymer for the photocatalytic activation of aryl halides
Source: Nat Commun. 2023 Jul 6;14:4002. doi: 10.1038/s41467-023-39540-z (PMC10326065; doi:10.1038/s41467-023-39540-z)

checkCIF/PLATON report

You have not supplied any structure factors. As a result the full set of tests cannot be run.

THIS REPORT IS FOR GUIDANCE ONLY. IF USED AS PART OF A REVIEW PROCEDURE FOR PUBLICATION, IT SHOULD NOT REPLACE THE EXPERTISE OF AN EXPERIENCED CRYSTALLOGRAPHIC REFEREE.

No syntax errors found. CIF dictionary Interpreting this report

Datablock: 1

Bond precision:	C-C = 0.0067 A	Wavelength=0.71073	
Cell:	a=22.9797(9)	b=22.7946(11)	c=18.5428(8)
	alpha=90	beta=122.783(1)	gamma=90
Temperature:	293 K		

	Calculated	Reported
Volume	8166.0(6)	8165.9(6)
Space group	C 2/c	C2/c
Hall group	-C 2yc	?
Moiety formula	2(C60 H20 Cd N4 O24), 2(C3 H6 N O), 4(C3 H7 N O), 8(C2 H7 N), 2	?
Sum formula	C154 H136 Cd2 N22 O57	C77 H68 Cd N11 O28.50
Mr	3431.67	1715.82
Dx,g cm-3	1.396	1.396
Z	2	4
Mu (mm-1)	0.355	0.355
F000	3532.0	3532.0
F000'	3530.70	
h,k,lmax	27,27,22	27,27,22
Nref	7182	7073
Tmin,Tmax	0.915,0.955	0.916,0.955
Tmin'	0.915	

Correction method= # Reported T Limits: Tmin=0.916 Tmax=0.955
AbsCorr = MULTI-SCAN

Data completeness= 0.985 Theta(max)= 25.000

R(reflections)= 0.0530(6131)	wR2(reflections)= 0.1519(7073)
S = 1.054 Npar= 541	

The following ALERTS were generated. Each ALERT has the format
test-name_ALERT_alert-type_alert-level.
Click on the hyperlinks for more details of the test.

Alert level C

```
PLAT199_ALERT_1_C Reported _cell_measurement_temperature ..... (K)        293 Check
PLAT200_ALERT_1_C Reported   _diffrn_ambient_temperature ..... (K)        293 Check
PLAT220_ALERT_2_C NonSolvent   Resd 1  O   Ueq(max)/Ueq(min) Range        4.1 Ratio
PLAT232_ALERT_2_C Hirshfeld Test Diff (M-X)  Cd1      --O2       .        5.9 s.u.
PLAT242_ALERT_2_C Low    'MainMol' Ueq as Compared to Neighbors of         C2 Check
PLAT243_ALERT_4_C High   'Solvent' Ueq as Compared to Neighbors of       C106 Check
PLAT244_ALERT_4_C Low    'Solvent' Ueq as Compared to Neighbors of         N7 Check
PLAT244_ALERT_4_C Low    'Solvent' Ueq as Compared to Neighbors of         N5 Check
PLAT244_ALERT_4_C Low    'Solvent' Ueq as Compared to Neighbors of       C201 Check
PLAT244_ALERT_4_C Low    'Solvent' Ueq as Compared to Neighbors of         N3 Check
PLAT260_ALERT_2_C Large Average Ueq of Residue Including       O14      0.172 Check
PLAT260_ALERT_2_C Large Average Ueq of Residue Including       O13      0.187 Check
PLAT309_ALERT_2_C Single Bonded Oxygen (C-O > 1.3 Ang) ..........         O7 Check
PLAT317_ALERT_2_C Too many H on C in C=N Moiety in Solvent/Ion ...       C105 Check
PLAT601_ALERT_2_C Unit Cell Contains Solvent Accessible VOIDS of .         75 Ang**3
```

Alert level G

```
PLAT004_ALERT_5_G Polymeric Structure Found with Maximum Dimension          3 Info
PLAT005_ALERT_5_G No Embedded Refinement Details Found  in the CIF     Please Do !
PLAT007_ALERT_5_G Number of Unrefined Donor-H Atoms .............           2 Report
PLAT045_ALERT_1_G Calculated and Reported Z Differ by a Factor ...      0.500 Check
PLAT066_ALERT_1_G Predicted and Reported Tmin&Tmax Range Identical          ? Check
PLAT083_ALERT_2_G SHELXL Second Parameter in WGHT  Unusually Large      19.50 Why ?
PLAT093_ALERT_1_G No s.u.'s on H-positions, Refinement Reported as      mixed Check
PLAT128_ALERT_4_G Alternate Setting for Input Space Group     C2/c       I2/a Note
PLAT300_ALERT_4_G Atom Site Occupancy of O14        Constrained at        0.5 Check
PLAT300_ALERT_4_G Atom Site Occupancy of O1W        Constrained at        0.5 Check
PLAT300_ALERT_4_G Atom Site Occupancy of O2W        Constrained at        0.5 Check
PLAT302_ALERT_4_G Anion/Solvent/Minor-Residue Disorder (Resd  2  )        20% Note
PLAT302_ALERT_4_G Anion/Solvent/Minor-Residue Disorder (Resd  6  )       100% Note
PLAT302_ALERT_4_G Anion/Solvent/Minor-Residue Disorder (Resd  7  )       100% Note
PLAT311_ALERT_2_G Isolated Disordered Oxygen Atom (No H's ?) .....        O1W Check
PLAT311_ALERT_2_G Isolated Disordered Oxygen Atom (No H's ?) .....        O2W Check
PLAT380_ALERT_4_G Incorrectly? Oriented X(sp2)-Methyl Moiety .....       C105 Check
PLAT710_ALERT_4_G Delete 1-2-3 or 2-3-4 Linear Torsion Angle ... #         36 Do !
              O4  -CD1 -C1  -C7     94.00  3.00   2_755   1_555   1_555   1_555
PLAT710_ALERT_4_G Delete 1-2-3 or 2-3-4 Linear Torsion Angle ... #         37 Do !
              O4  -CD1 -C1  -C7   -143.00  3.00   1_555   1_555   1_555   1_555
PLAT710_ALERT_4_G Delete 1-2-3 or 2-3-4 Linear Torsion Angle ... #         38 Do !
              O1  -CD1 -C1  -C7    -50.00  3.00   2_755   1_555   1_555   1_555
PLAT710_ALERT_4_G Delete 1-2-3 or 2-3-4 Linear Torsion Angle ... #         39 Do !
              O1  -CD1 -C1  -C7     92.00  3.00   1_555   1_555   1_555   1_555
PLAT710_ALERT_4_G Delete 1-2-3 or 2-3-4 Linear Torsion Angle ... #         40 Do !
              O2  -CD1 -C1  -C7    -90.00  3.00   1_555   1_555   1_555   1_555
```

```
PLAT710_ALERT_4_G Delete 1-2-3 or 2-3-4 Linear Torsion Angle ... #         41 Do !
              O2  -CD1 -C1  -C7      4.00  3.00   2_755   1_555   1_555   1_555
PLAT710_ALERT_4_G Delete 1-2-3 or 2-3-4 Linear Torsion Angle ... #         42 Do !
              C1  -CD1 -C1  -C7    -22.00  3.00   2_755   1_555   1_555   1_555
PLAT710_ALERT_4_G Delete 1-2-3 or 2-3-4 Linear Torsion Angle ... #         61 Do !
              CD1 -C1  -C7  -C6    -82.00  3.00   1_555   1_555   1_555   1_555
PLAT710_ALERT_4_G Delete 1-2-3 or 2-3-4 Linear Torsion Angle ... #         64 Do !
              CD1 -C1  -C7  -C8     96.00  3.00   1_555   1_555   1_555   1_555
PLAT794_ALERT_5_G Tentative Bond Valency for Cd1       (II)      .       1.92 Info
PLAT899_ALERT_4_G SHELXL-97  is Deprecated and Succeeded by SHELXL     2019/3 Note
```

```
   0 ALERT level A = Most likely a serious problem - resolve or explain
   0 ALERT level B = A potentially serious problem, consider carefully
  15 ALERT level C = Check. Ensure it is not caused by an omission or oversight
  28 ALERT level G = General information/check it is not something unexpected

   5 ALERT type 1 CIF construction/syntax error, inconsistent or missing data
  11 ALERT type 2 Indicator that the structure model may be wrong or deficient
   0 ALERT type 3 Indicator that the structure quality may be low
  23 ALERT type 4 Improvement, methodology, query or suggestion
   4 ALERT type 5 Informative message, check
```

It is advisable to attempt to resolve as many as possible of the alerts in all categories. Often the minor alerts point to easily fixed oversights, errors and omissions in your CIF or refinement strategy, so attention to these fine details can be worthwhile. In order to resolve some of the more serious problems it may be necessary to carry out additional measurements or structure refinements. However, the purpose of your study may justify the reported deviations and the more serious of these should normally be commented upon in the discussion or experimental section of a paper or in the "special_details" fields of the CIF. checkCIF was carefully designed to identify outliers and unusual parameters, but every test has its limitations and alerts that are not important in a particular case may appear. Conversely, the absence of alerts does not guarantee there are no aspects of the results needing attention. It is up to the individual to critically assess their own results and, if necessary, seek expert advice.

Publication of your CIF in IUCr journals

A basic structural check has been run on your CIF. These basic checks will be run on all CIFs submitted for publication in IUCr journals (*Acta Crystallographica*, *Journal of Applied Crystallography*, *Journal of Synchrotron Radiation*); however, if you intend to submit to *Acta Crystallographica Section C* or *E* or *IUCrData*, you should make sure that full publication checks are run on the final version of your CIF prior to submission.

Publication of your CIF in other journals

Please refer to the *Notes for Authors* of the relevant journal for any special instructions relating to CIF submission.

PLATON version of 10/05/2023; check.def file version of 10/05/2023

Datablock 1 - ellipsoid plot

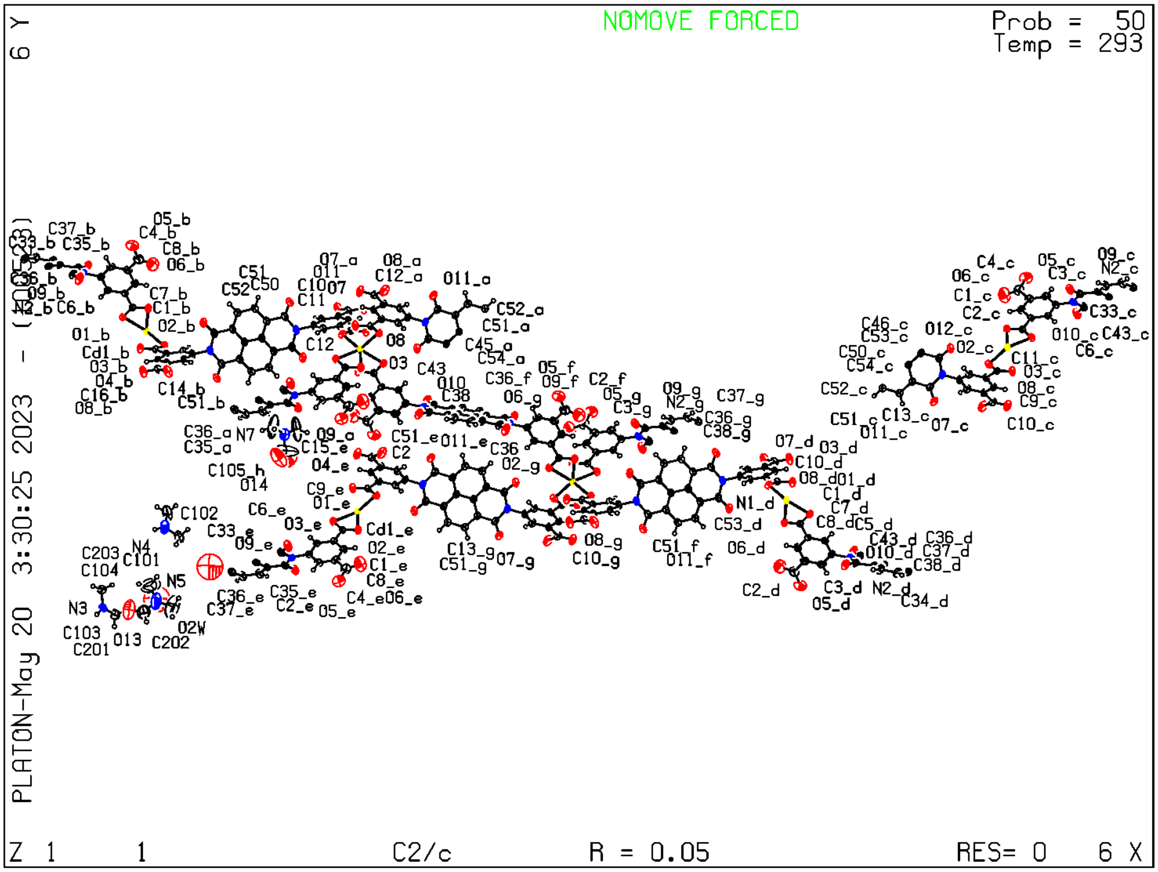

Supplement: Supplementary file 3 — Supplementary Data 1-3 [file 41467_2023_39540_MOESM3_ESM.zip › Supplementary Data 2-checkCIF of Cd-NDI.pdf]
