# Supplementary material for: Chalcogen-bridged coordination polymer for the photocatalytic activation of aryl halides
Source: Nat Commun. 2023 Jul 6;14:4002. doi: 10.1038/s41467-023-39540-z (PMC10326065; doi:10.1038/s41467-023-39540-z)

checkCIF/PLATON report

You have not supplied any structure factors. As a result the full set of tests cannot be run.

THIS REPORT IS FOR GUIDANCE ONLY. IF USED AS PART OF A REVIEW PROCEDURE FOR PUBLICATION, IT SHOULD NOT REPLACE THE EXPERTISE OF AN EXPERIENCED CRYSTALLOGRAPHIC REFEREE.

No syntax errors found. CIF dictionary Interpreting this report

Datablock: 1

```
Bond precision:  C-C = 0.0045 A          Wavelength=0.71073

Cell:            a=28.3250(11)     b=28.3250(11)     c=13.7766(5)
                 alpha=90          beta=90           gamma=90
Temperature:     150 K
```

	Calculated	Reported
Volume	11053.1(9)	11053.0(9)
Space group	I 41/a	I 41/a
Hall group	-I 4ad	-I 4ad
Moiety formula	C21 H19 N3 O8 Sr, C8 H5	?
Sum formula	C29 H24 N3 O8 Sr	C3.41 H2.82 N0.35 O0.94 Sr0.12
Mr	630.13	74.13
Dx,g cm-3	1.515	1.515
Z	16	136
Mu (mm-1)	2.007	2.007
F000	5136.0	5136.0
F000’	5113.97	
h,k,lmax	33,33,16	33,33,16
Nref	4868	4864
Tmin,Tmax		
Tmin’		

```
Correction method= Not given

Data completeness= 0.999          Theta(max)= 24.998

R(reflections)= 0.0262( 4500)     wR2(reflections)= 0.0627( 4864)

S = 1.079                   Npar= 375
```

The following ALERTS were generated. Each ALERT has the format
test-name_ALERT_alert-type_alert-level.
Click on the hyperlinks for more details of the test.

Alert level C

```
ABSMU01_ALERT_1_C  The ratio of given/expected absorption coefficient lies
               outside the range 0.99 <> 1.01
            Calculated value of mu =     2.045
            Value of mu given      =     2.007
PLAT053_ALERT_1_C Minimum Crystal Dimension Missing (or Error) ...     Please Check
PLAT054_ALERT_1_C Medium  Crystal Dimension Missing (or Error) ...     Please Check
PLAT055_ALERT_1_C Maximum Crystal Dimension Missing (or Error) ...     Please Check
PLAT220_ALERT_2_C NonSolvent   Resd 1  C   Ueq(max)/Ueq(min) Range        3.6 Ratio
PLAT222_ALERT_3_C NonSolvent Resd 1  H   Uiso(max)/Uiso(min) Range        4.4 Ratio
```

Alert level G

```
CELLZ01_ALERT_1_G Difference between formula and atom_site contents detected.
CELLZ01_ALERT_1_G ALERT: Large difference may be due to a
            symmetry error - see SYMMG tests
           From the CIF: _cell_formula_units_Z  136
           From the CIF: _chemical_formula_sum  C3.41 H2.82 N0.35 O0.94 Sr0.12
           TEST: Compare cell contents of formula and atom_site data

           atom    Z*formula  cif sites diff
           C        463.76    464.00   -0.24
           H        383.52    384.00   -0.48
           N         47.60     48.00   -0.40
           O        127.84    128.00   -0.16
           Sr        16.32     16.00    0.32
PLAT004_ALERT_5_G Polymeric Structure Found with Maximum Dimension          3 Info
PLAT045_ALERT_1_G Calculated and Reported Z Differ by a Factor ...       0.12 Check
PLAT083_ALERT_2_G SHELXL Second Parameter in WGHT  Unusually Large       7.57 Why ?
PLAT774_ALERT_1_G Check   X-Y Bond in CIF: Sr1      --Sr1       ..       4.25 Ang.
PLAT774_ALERT_1_G Check   X-Y Bond in CIF: Sr1      --Sr1       ..       4.25 Ang.
PLAT870_ALERT_4_G ALERTS Related to Twinning Effects Suppressed ..          ! Info
PLAT883_ALERT_1_G No Info/Value for _atom_sites_solution_primary .     Please Do !
```

---

0 **ALERT level A** = Most likely a serious problem - resolve or explain
0 **ALERT level B** = A potentially serious problem, consider carefully
6 **ALERT level C** = Check. Ensure it is not caused by an omission or oversight
9 **ALERT level G** = General information/check it is not something unexpected

10 ALERT type 1 CIF construction/syntax error, inconsistent or missing data
2 ALERT type 2 Indicator that the structure model may be wrong or deficient
1 ALERT type 3 Indicator that the structure quality may be low
1 ALERT type 4 Improvement, methodology, query or suggestion
1 ALERT type 5 Informative message, check

It is advisable to attempt to resolve as many as possible of the alerts in all categories. Often the minor alerts point to easily fixed oversights, errors and omissions in your CIF or refinement strategy, so attention to these fine details can be worthwhile. In order to resolve some of the more serious problems it may be necessary to carry out additional measurements or structure refinements. However, the purpose of your study may justify the reported deviations and the more serious of these should normally be commented upon in the discussion or experimental section of a paper or in the "special_details" fields of the CIF. checkCIF was carefully designed to identify outliers and unusual parameters, but every test has its limitations and alerts that are not important in a particular case may appear. Conversely, the absence of alerts does not guarantee there are no aspects of the results needing attention. It is up to the individual to critically assess their own results and, if necessary, seek expert advice.

Publication of your CIF in IUCr journals

A basic structural check has been run on your CIF. These basic checks will be run on all CIFs submitted for publication in IUCr journals (*Acta Crystallographica*, *Journal of Applied Crystallography*, *Journal of Synchrotron Radiation*); however, if you intend to submit to *Acta Crystallographica Section C* or *E* or *IUCrData*, you should make sure that full publication checks are run on the final version of your CIF prior to submission.

Publication of your CIF in other journals

Please refer to the *Notes for Authors* of the relevant journal for any special instructions relating to CIF submission.

PLATON version of 05/12/2020; check.def file version of 05/12/2020

Datablock 1 - ellipsoid plot

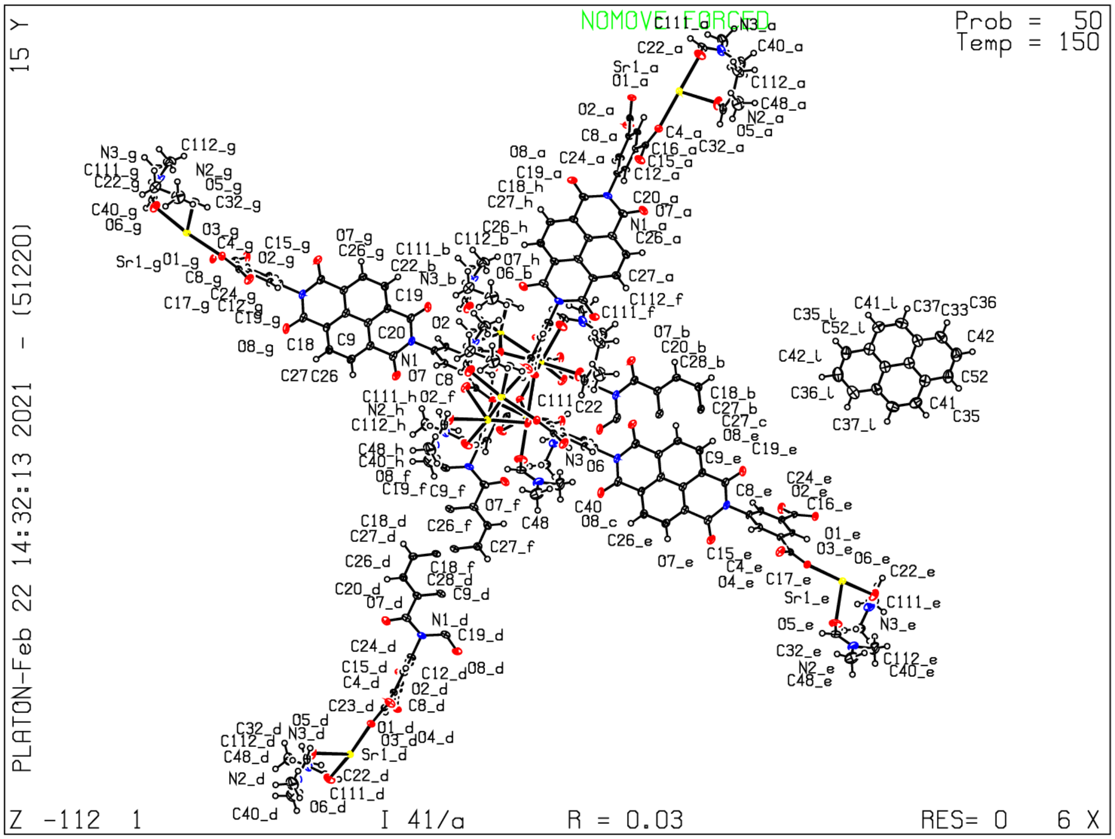

Supplement: Supplementary file 3 — Supplementary Data 1-3 [file 41467_2023_39540_MOESM3_ESM.zip › Supplementary Data 3-checkCIF of Pyrene@Sr-NDI.pdf]
